# Deciding to simulate: Cognitive mechanisms of predicting the decisions of others

**DOI:** 10.1016/j.isci.2026.116047

**Published:** 2026-06-24

**Authors:** Erik Stuchlý, Sophie Bavard, Sebastian Gluth

**Affiliations:** 1 Department of Psychology and Hamburg Center of Neural and Cognitive Systems, University of Hamburg, Von-Melle Park 11, 20146 Hamburg, Germany; 2 Paris Brain Institute - ICM, Hôpital de la Pitié-Salpêtrière, 47 Boulevard de l’Hôpital, 75013 Paris, France

**Keywords:** Neuroscience, Behavioral neuroscience, Cognitive neuroscience

## Abstract

Existing behavioral and neural evidence suggests that people rely on mental simulation when predicting others’ decisions. If true, making predictions with a biased decision system should result in biased predictions. We tested this idea directly by biasing participants’ risk valuation through adaptation to either high- or low-probability contexts and then asking them to predict the choices of three distinct agents (risk-average, risk-averse, risk-seeking). The adaptation manipulation biased participants’ predictions for the risk-average and risk-averse agents, suggesting simulation with their own (biased) decision process when predicting these agents. In contrast, there was no adaptation effect for the risk-seeking agent. Similarly, participants’ own risk tendencies correlated with predictions for the risk-average and risk-averse, but not the risk-seeking agent. Drift diffusion modeling analyses showed that this adaptation bias was the result of changes in participants’ risk valuation parameter. Overall, these findings support simulation-based prediction while suggesting a modulating role of the other person’s characteristics.

## Highlights

• Biased decision process leads to biased predictions of others’ decisions
• Modeling revealed risk perception to underlie this bias in decisions and predictions
• Decision bias does not transfer to predictions of agents with certain characteristics

## Graphical abstract

**Figure:**
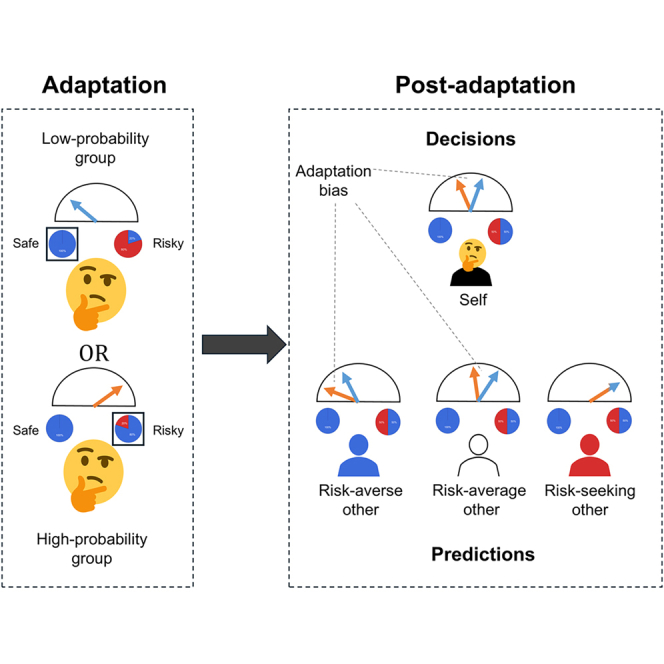

## Introduction

Consider the following situation: you meet up with a new friend and propose either grabbing a fast-food meal or going to a salad bar for lunch. Your friend’s decision to eat a salad will allow you to infer some information about their current preference—specifically, that they prefer a healthier salad over (potentially tastier) junk food. Knowing this, chances are you would be able to make an informed prediction about their choice of drink later on—for instance, that they might choose a healthy smoothie over a Coke, even if you yourself might prefer a sweet, fizzy beverage. This sophisticated prediction is possible due to the fundamental understanding that other people have mental and cognitive states separate from our own, and that these states guide behavior, referred to as Theory of Mind (ToM).^1^ Contemporary accounts agree that predicting others’ behavior relies on a mixture of using general knowledge about human behavior, and of mentally simulating the other person’s purported state of mind.^2^^,^^3^^,^^4^ Likewise, most accounts agree that, to successfully understand the behavior of others, individuals need to possess a model of how the behavior is generated; this model can then be inverted to infer the mental state from observed behavior.^5^^,^^6^

Despite the fundamental cognitive^7^^,^^8^ and neural^9^^,^^10^ mechanisms underlying decision-making for the self being relatively well-explored, how exactly predictions of others’ decisions are achieved is relatively understudied. Multiple strands of evidence, however, suggest that there is a mechanistic overlap between the two types of decisions. For instance, individuals use their own preferences as a prior when learning the preferences of others and predicting their choices,^11^ especially if the other person is similar to oneself.^12^ Even when the other individual’s preferences have been learned, predictions of their decisions often remain biased by one’s own preference.^13^^,^^14^ Crucially, predicted decisions mimic the response times that would be expected based on the other person’s preferences, even if one’s own preferences are different and would thus lead to different response times.^15^ This suggests that people truly engage in the decision process from the other person’s perspective. Such findings are in agreement with neuroimaging studies showing that option value during prediction is not encoded according to the participant’s own, but rather the other person’s preference.^16^^,^^17^^,^^18^ Current literature thus shows a mechanistic overlap in choices for oneself and predicted decisions of others, consistent with a simulation account whereby people make predicted decisions by simulating them with their own decision-making process.

In the present study, we sought to experimentally verify the simulation hypothesis and test whether people engage their own valuation system when predicting the choice of others. To this end, we leveraged the fact that the value of decision options is not determined in isolation, but is affected by the wider context within which it occurs.^19^^,^^20^^,^^21^^,^^22^ Specifically, exposure and adaptation to a previous context can alter the way a subsequently presented option’s value is perceived.^23^ For instance, if participants become adapted to a context with high-value options on average, they will perceive subsequently presented intermediate-value options as less appealing than when they are first adapted to a context with low-value options.^24^ Such adaptation to average option value can also be observed in risky choice^25^ and intertemporal choice,^26^ pointing to a general mechanism across decision domains, often attributed to exposure-related shift in participants’ reference points. Importantly, it can be assumed that people are not aware of these biases, otherwise they could simply suppress them to make more rational choices.^27^ Therefore, if adapting participants to a specific decision context resulted in a valuation bias when subsequently predicting other people’s decisions, it would support the idea that the predictions are carried out by participant’s own (biased) valuation system.^28^

To rigorously test this idea, we conducted three pre-registered online experiments using a risky choice paradigm, where we first biased participant’s valuation process in the “adaptation stage” (by splitting them into two groups with gambles of predominantly high- vs. low-probability of obtaining a reward), and subsequently observed whether a choice bias emerges when they make decisions in the “post-adaptation stage.” The control experiment was conducted as a baseline, checking whether the bias appears when participants keep making decisions for themselves after adaptation (i.e., whether the group previously exposed to high-probability gambles becomes less likely to accept medium-probability gambles than the group previously exposed to low-probability gambles). Experiment 1 was conducted to test whether the valuation bias persists when participants predict the behavior of a (hypothetical) non-specific, average other person. Crucially, to firmly rule out the possibility that participants choose for themselves when making predictions, experiment 2 tested whether the valuation bias manifests when predicting the choices of two hypothetical agents with defined, extreme risk preferences. To better understand whether and how the valuation bias differs as a function of who the decision is made for (e.g., whether the group difference in choices is captured by a shifted risk preference, or an automatic bias toward accepting/rejecting the risky option), we also fitted the data from each post-adaptation stage with an evidence accumulation model, which decomposes decisions into fundamental mechanisms such as strength of valuation, decision caution or initial bias.^29^^,^^30^ Our findings generally provide support for the main hypothesis; we found that the adaptation manipulation results in biased predictions, suggesting that these predictions were made with participants’ own (biased) decision-making system. However, this effect only emerged during predictions for the average and risk-averse, but not risk-seeking agent, suggesting that the other agent’s characteristics may determine the extent to which one’s own decision system is utilized.

## Results

### Behavioral results

**Figure 1:**
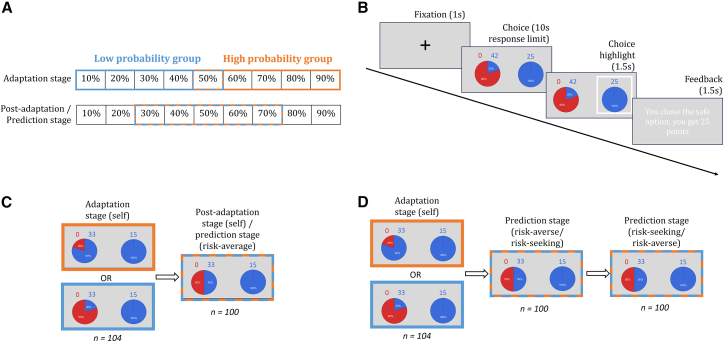
Design of the experiments Note: (A) Range of probabilities of obtaining the non-zero outcome from the risky option, in each experimental stage, for both the high-probability (orange) and low-probability (blue) groups. (B) Time-course of a single trial. (C) Graphical representation of the two stages of the control experiment and experiment 1. (D) Graphical representation of the three stages of experiment 2. The order of the prediction stages was counter-balanced across participants.

**Figure 2:**
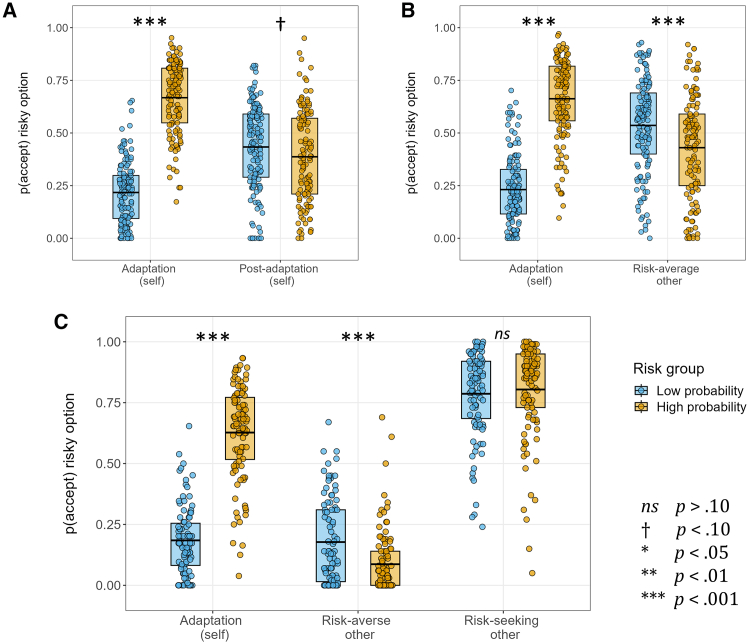
Group-level mean acceptance rate Note: Mean acceptance rates of the risky option across conditions and stages from the control experiment (A), experiment 1 (B), and experiment 2 (C). Each point in the graphs represents the mean of a single participant, with the boxes denoting the population means and the 25th and 75th quantiles of the distribution.

The task consisted of making risky choice decisions between a “safe” option with a guaranteed but small payoff, and a “risky” option with a certain probability of obtaining a larger payoff, otherwise gaining nothing). In the first “adaptation” stage, we manipulated the distribution of expected utilities of the risky option, such that one group of participants (high-probability group, HP) made decisions in a context where the risky options were preferable to the safe option on average, whereas the other group (low-probability group, LP) made decisions in a context with less preferable risky options (Figure 1A, Table 1). In the post-adaptation stage, participants made decisions for themselves (control experiment; Figure 1C)/predicted the decision of another agent (risk-average other in experiment 1; risk-averse and risk-seeking others in experiment 2, in a counter-balanced order; Figure 1D), within a context where the expected utilities of the safe and risky options were balanced, on average. Figure 2 summarizes the distribution of the mean probability of accepting the risky option $\left(p_{\mathrm{accept}}\right)$—the main behavioral measure of interest—across all groups and conditions, for all three experiments. The aim of the three experiments was to check whether the adaptation manipulation works in inducing a bias when making decisions for the self (control experiment) and whether this bias also transfers to predictions made for a vaguely specified other (experiment 1) and for others with specific preferences (experiment 2).

**Table 1:** Value differences across different experimental stages

Condition	Subjective value differences						
Adaptation LP	−4.5	−3	**−1.5**	**0**	**1.5**		
Adaptation HP			**−1.5**	**0**	**1.5**	3	4.5
Post-adaptation		−3	**−1.5**	**0**	**1.5**	3	

LP refers to a low probability, HP to a high probability condition. Negative VD indicates a higher value of the safe option, whereas positive VD indicates a higher value of the risky option. Bolded highlights show the trial types that overlapped across all the experimental conditions.

First, we focus on results from the adaptation stage of all experiments, where we expected the HP/LP group to accept the risky option on 75%/25% of trials if participants displayed typical distortions in value and probability representation. The mean acceptance rates were higher than chance (50%) in the HP group (t(125) = 11.14, p< 0.001 control experiment; t(128) = 9.51, p< 0.001 experiment 1; t(103) = 6.69, p< 0.001 experiment 2), and lower than chance in LP group (t(139) = −22.08, p< 0.001 control experiment; t(136) = −18.96, p< 0.001 experiment 1; t(94) = −22.07, p< 0.001 experiment 2). On average, participants were more risk-averse than we anticipated, with the $p_{\mathrm{accept}}$ rates being lower than 75% in the HP group (t(125) = −5.49, p< 0.001 control experiment; t(128) = −5.12, p< 0.001 experiment 1; t(103) = −6.45, p< 0.001 experiment 2) and lower than 25% in the LP group (t(139) = −2.63, p= 0.009 control experiment; t(94) = −4.58, p< 0.001 experiment 2), the only exception being the LP group in experiment 1 (t(136) = −1.33, p= 0.185). Importantly though, as the post-hoc tests indicate, HP individuals clearly accepted the risky option more often than LP individuals in the control experiment (t(264) = 23.27, p< 0.001), experiment 1 (t(264) = 19.51, p< 0.001) and experiment 2 (t(197) = 18.34, p< 0.001), meaning that the manipulation produced the desired behavior.

**Figure 3:**
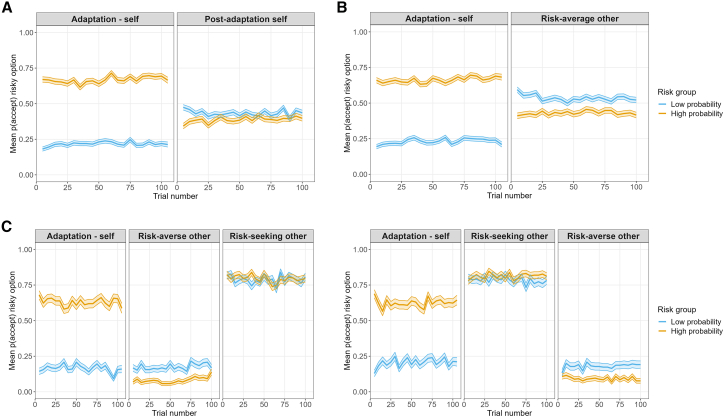
Trial-level acceptance rates Note: Trial-level mean $p_{\mathrm{accept}}$ (thick line) across all participants and its standard error (colored ribbon), split across probability groups for (A) control experiment, (B) experiment 1, and (C) experiment 2 (participants who first predicted for the risk-averse agent appear on the left and participants who first predicted for the risk-seeking agent appear on the right). For better readability, a smoothing was applied to the plotted trace-plots (but not the statistical analyses), such that each “dent” in the line corresponds to a moving average of 5 consecutive trials.

Turning to the main research question of whether the adaptation manipulation induced a group difference in post-adaptation $p_{\mathrm{accept}}$, a 2-by-2 mixed repeated measures ANOVA on the data from the control experiment showed a significant main effect of probability group (F(1,264) = 90.80, p< 0.001, $\eta_{G}^{2}$ = 0.226), stage (F(1,264) = 13.20, p< 0.001, $\eta_{G}^{2}$ = 0.007) and probability group-by-stage interaction (F(1,264) = 783.43, p< 0.001, $\eta_{G}^{2}$ = 0.307). Crucially, we found the expected trend in the post-adaptation stage, whereby the LP mean was higher than the HP mean; however, this difference was only approaching significance (t(264) = 1.781, p = 0.076). To inspect this pattern more closely, we conducted an exploratory analysis of trial-level mean $p_{\mathrm{accept}}$ throughout the entire experiment, separately for HP and LP participants. Figure 3A displays this traceplot for the control experiment. At the beginning of the post-adaptation stage, an effect consistent with the valuation bias can be seen, whereby LP participants initially have a higher mean $p_{\mathrm{accept}}$ than HP participants, until they converge later in the stage. Fitting an exponential decay model (Appendix C) confirmed that there was a gradual change in choice behavior over trials. Altogether, the adaptation manipulation was effective in inducing the anticipated valuation bias when participants decided for themselves, albeit less strongly than expected.

To test our central hypothesis that a similar valuation bias will emerge when making predictions for someone else, we examined data from experiment 1. A 2-by-2 (probability group) mixed repeated measures ANOVA showed a significant main effect of probability group (F(1,264) = 63.44, p< 0.001, $\eta_{G}^{2}$ = 0.133) and probability group-by-stage interaction (F(1,264) = 303.43, p< 0.001, $\eta_{G}^{2}$ = 0.294); however, no main effect of stage (F(1,264) = 5.51, p = 0.020, $\eta_{G}^{2}$ = 0.008). Importantly, in the average other condition, LP group accepted the risky option significantly more than the HP group (t(1,264) = 3.69, p< 0.001). Figure 3B shows that the trial-by-trial post-adaptation pattern is qualitatively similar to that in the control experiment, with the group difference being largest at the very beginning of the stage and then decreasing (as confirmed by the exponential decay model analysis in Appendix C). However, the adaptation clearly produced a stronger effect in this experiment, suggesting that people may be even more prone to such bias when predicting another person’s decision.

Finally, in experiment 2, a 3-by-2 mixed repeated measures ANOVA revealed a significant main effect of probability group (F(1,197) = 67.42, p< 0.001, $\eta_{G}^{2}$ = 0.117), stage (F(1.77,349.59) = 829.04, p< 0.001, $\eta_{G}^{2}$ = 0.721) and their interaction (F(1.77,349.59) = 148.48, p< 0.001, $\eta_{G}^{2}$ = 0.316). Importantly, the LP group predicted higher $p_{\mathrm{accept}}$ than the HP group for the risk-averse agent (t(197)=4.187, p< 0.001). On the other hand, the group difference in the risk-seeking prediction stage was not statistically significant (t(197)=0.669, p=.504). Similar to the post-adaptation stage of the control experiment, we inquired whether an initial group difference in the risk-seeking prediction stage might have emerged immediately after the adaptation stage but dissipated over the course of the block. If true, we would additionally expect that any such post-adaptation effect should be present mainly when the risk-seeking other stage was encountered immediately after the adaptation stage (rather than after the risk-averse other stage). To this end, we plotted the same trial-level mean $p_{\mathrm{accept}}$ trace-plots as before, separately for participants who first predicted the risk-averse/risk-seeking agent (Figure 3C) and conducted a 2 (order) by 2 (agent) by 2 (probability group) ANOVA on the prediction data. We did not find qualitative evidence of the adaptation effect being strong at the beginning of the stage and disappearing throughout its duration (neither for risk-averse nor for risk-seeking agents). Remarkably, whether a particular agent was encountered first or second had no bearing on the presence or absence of the adaptation effect as indicated by a non-significant main effect of order (F(1,195) = 0.27, p= 0.603, $\eta_{G}^{2}<$ 0.001), and non-significant interactions between order and agent (F(1,195) = 0.01, p= 0.983, $\eta_{G}^{2}$ = < 0.001) as well as between order, agent and probability group (F(1,195) = 0.04, p= 0.845, $\eta_{G}^{2}$ = 0.040). An exponential decay analysis confirmed that there was no substantial change in choice behavior over trials for either agent, regardless of the order of presentation (Appendix C). Overall, these results indicate a stable between-group difference in predictions for the risk-averse agent, and an equally consistent lack of difference in predictions for the risk-seeking agent. More generally, the post-adaptation effect was apparent in most experiments, but with qualitative and quantitative differences depending on the characteristics of those for whom the decisions were made.

**Figure 4:**
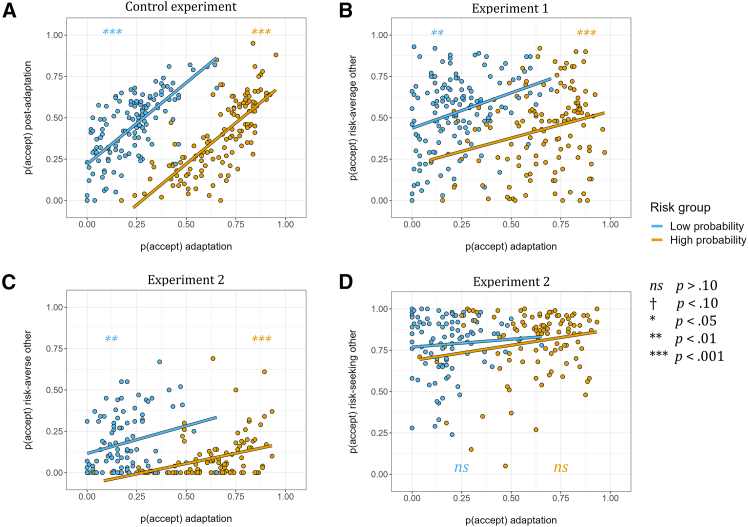
Acceptance rate correlations across experimental stages Note: Correlation plots show the relationship between $p_{\mathrm{accept}}$ rates in the adaptation stage (x axis) and the post-adaptation stage (y axis). Each point represents the mean data for a single participant, alongside the line of best fit for each group. (A) Control experiment. (B) Experiment 1. (C) Experiment 2 risk-averse condition. (D) Experiment 2 risk-seeking condition.

Given that we found the expected adaptation effects in terms of biased predictions when participants predicted the risk-average and risk-averse agents, but not when predicting the risk-seeking agent, the results so far indicate that individuals might rely on their own (biased) decision-making system only when making predictions for the former but not the latter agents. Therefore, we sought to further explore the association between participants’ own decision behavior and their predictions, separately for the different agents (hypothesis 3). Figure 4 shows the correlations between $p_{\mathrm{accept}}$ in the adaptation stage (i.e., decisions for themselves) and the predicted $p_{\mathrm{accept}}$ in the post-adaptation stage across all three experiments. Expectedly, the correlation in the control experiment is highly positive in both HP (ρ(125) = 0.779, p< 0.001) and LP (ρ(139) = 0.698, p< 0.001) groups, showing that participants themselves exhibited stable risk preferences. A similar, albeit weaker association in $p_{\mathrm{accept}}$ between stages was found in experiment 1 HP (ρ(128) = 0.237, p = 0.007) and LP (ρ(136) = 0.289, p< 0.001) groups. Inspecting the association between self and risk-averse other conditions in experiment 2, we also observe a positive, statistically significant relationship in the HP group (ρ(103) = 0.416, p< 0.001) and the LP group (ρ(94) = 0.294, p = 0.004). On the other hand, the relationship between decision behavior in the self and risk-seeking other conditions failed to reach statistical significance, both in the HP (ρ(103) = 0.086, p = 0.384) and the LP group (ρ(94) = 0.010, p = 0.922). A Fisher’s z-test confirmed that the correlation between self and risk-seeking other conditions is weaker than between self and risk-averse other in HP (z = 2.33, p = 0.010) and LP (z = 1.67, p = 0.048) groups, and also significantly weaker than the correlation between self and risk-average other in the LP group (z = 2.12, p = 0.017)—though not the HP group (z = 1.16, p = 0.122). Thus, participants’ own risk tendency was positively linked to their predictions of risk-average and risk-averse, but not risk-seeking agents, providing further evidence that they did not rely on their own decision-making system to the same extent when predicting the behavior of the risk-seeking agent. This difference, however, cannot be solely explained by a higher average similarity with the risk-averse than the risk-seeking agent (Appendix D), suggesting that the reliance on one’s own decision-making system is likely modulated by multiple facets of the other’s identity.

### Modeling results

To gain a mechanistic understanding of the observed adaptation effects, we fitted the data with a modified drift diffusion model (DDM), which assumes that the rate of evidence accumulation on any particular trial is given by the scaled subjective value difference (sVD) between the safe and risky option, with sVD being computed in line with Prospect Theory (PT-DDM; see Methods for more details and Table 2 for the list of all parameters). Because the behavioral adaptation effect was not qualitatively and quantitatively the same across the experiments, investigating the difference in PT-DDM parameters between the HP and LP stage in each experiment could be informative as to which processes contributed to these differences.

**Table 2:** Glossary of symbols and abbreviations

Symbol	Description
sVD	subjective value difference between the two options
OV	overall value of the two options
α	magnitude discounting/risk valuation parameter
γ	probability distortion parameter
τ	boundary separation parameter
ds	drift scaling parameter
ndt	nondecision time parameter
sp	starting point bias parameter

First, we compared the fit of this model to three other versions (see Methods): One that assumed that the rate of evidence accumulation is given either by the difference between the options’ expected utility (EU-DDM), or their expected values (EV-DDM). The third alternative model assumed that participants ignore trial-level information and instead use a single drift rate across trials, leading to a probability-matching-like strategy which aims for a certain proportion of accepted risky options (heur-DDM). This model was included to check whether participants consider the available options in all conditions, instead of using a simple strategy that mimics risk-averse or risk-taking behavior by allocating risky choices in line with probability-matching. Because the PT-DDM provided the best fit in most conditions (Table S5 in Appendix F), all subsequent analyses were carried out with this model. Importantly, the heur-DDM did not provide the best fit to data from any condition, showing that participants accumulated available trial-level information in all stages. We additionally confirmed that if the responses had been generated with such a heuristic strategy (i.e., simulated with the heur-DDM), the heur-DDM would provide a better fit than the PT-DDM (see Appendix F for more detail).

**Figure 5:**
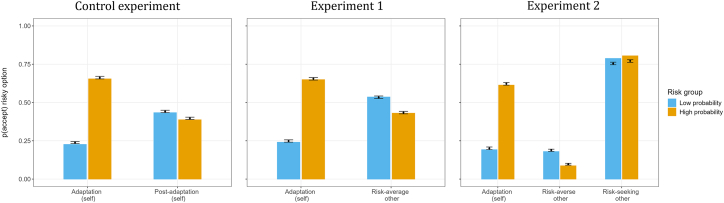
Posterior predictive check of the PT-DDM for choice data from all experimental conditions Note: The bars show the real mean group-level choice data in each experimental stage. The super-imposed black points with whiskers show the mean and 95% HDI of the mean choices obtained from 500 simulated datasets, all generated with the best-fitting parameter values in that condition (i.e., the posterior draw with the highest negative log likelihood value was used to determine the participant-level parameters used for simulations).

To assess the validity of the PT-DDM and its estimated parameter values (Table S7 in Appendix F), we performed posterior predictive checks to compare model-generated data against real data. Figure 5 shows the real group-level mean $p_{\mathrm{accept}}$ data from each post-adaptation stage of the three experiments, compared against the corresponding group-level mean $p_{\mathrm{accept}}$ distributions from the simulated posterior predictive datasets. In most conditions, the group-level means fell within or very close to the 95% HDI of the distribution of simulated means, meaning the model captured the choice data well. Importantly, the main pattern of differences (or their lack thereof) between HP and LP groups is preserved even in the simulated posterior predictive means. As such, the PT-DDM provides a good account of the qualitative trends in the group-level behavioral data and the underlying process behind both decisions for self and predicted decisions (for posterior predictives for response times on the group and participant levels, please refer to Appendix F).

**Figure 6:**
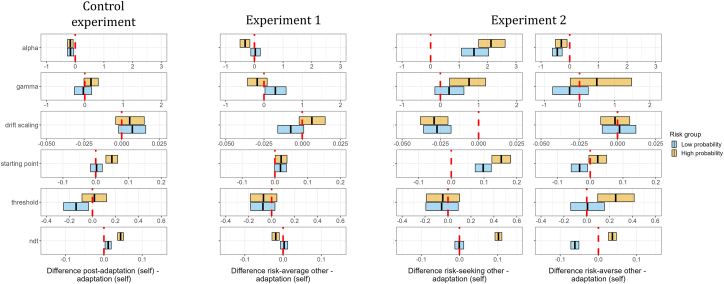
Difference in group-level PT-DDM parameters between HP and LP groups in the post-adaptation stages Note: The thick line represents the mean of this difference, with the edges of the bars representing the 95% HDI of this distribution of difference. For practical purposes, we consider the difference to be meaningfully large if the 95% HDI does not include 0 (the dashed red line). Distributions located to the left of zero (negative values) indicate that the particular parameter had a higher value in the HP group, whereas distributions to the right (positive side) indicate a higher parameter value in the LP group.

We then computed the between-group difference in posterior parameter values in all post-adaptation stages, to investigate how the PT-DDM accounts for valuation bias during choice for self (control experiment), and whether the same parameter differences are also observed in the prediction stages of experiments 1 and 2. As Figure 6 shows, in the post-adaptation stage of the control experiment, the LP group had both a higher α (HDI=[0.074,0.270])—representing a shift in risk valuation toward more risk-seeking behavior—and a lower sp (HDI=[−0.067,−0.031])—representing an automatic response bias toward choosing the safe option regardless of the options’ current value. Interestingly, the impact of the starting point bias difference on behavior (lower $p_{\mathrm{accept}}$ in the LP group) goes in the opposite direction of the impact of α difference (higher $p_{\mathrm{accept}}$ in the LP group). This suggests that the adaptation effect produces two distinct effects—one on option valuation being contrasted against values in the previous context, and one on the tendency to keep accepting the option that was chosen more often in the previous context.^31^ This conflict may help explain why the adaptation effect in the control experiment was relatively weak.

The pattern of differences in the average-other prediction stage was nearly identical, with the LP group showing higher α (HDI=[0.158,0.502]) and lower sp (HDI=[−0.037,−0.001]) parameter values. It is noteworthy that, relative to the control experiment, the α difference is larger, while the sp difference is smaller, again corresponding to the behavioral adaptation effect being stronger in experiment 1. A similar risk valuation difference can be observed in experiment 2, risk-averse predictions, with the LP group showing higher α (HDI=[0.093,0.498]) values. The difference in the sp parameter (HDI=[−0.050,0.004]) was not large enough to be credible, although its magnitude is similar to that in experiment 1. Interestingly, the τ parameter was also lower in the LP group (HDI=[−0.415,−0.096]), representing lower caution when making predictions for this agent. This indicates that the adaptation bias emerged via additional mechanisms during these predictions, which could have contributed to the behavioral effect being qualitatively different in this condition (i.e., no change in choice behavior across trials). Within the risk-seeking agent predictions, the sp has a similar (non-credible) magnitude of difference as in the other prediction stages (HDI=[−0.051,0.005]), indicating that by itself, this mechanism cannot explain the adaptation-induced effect on choices. Importantly, we see a lack of differences in other parameters, particularly with α not differing between the two groups (HDI=[−0.344,0.331]). This further confirms that the adaptation bias has not translated to these predictions. We note that, in all experiments, we observe group differences in the ndt parameter; however, since it only influences response times and not choices, its involvement in the valuation bias is less informative (moreover, as Figure S6 in Appendix F shows, this parameter recovers less reliably than others, making the differences difficult to interpret).

In summary, the most consistent difference across stages was in the α parameter, the magnitude of which qualitatively corresponded to the strength of the adaptation effect on choice across the experiments (or, in the case of risk-seeking other predictions, the lack of effect). The adaptation stage also induced a perseverance-like choice predisposition in decisions for oneself, which translated into predictions only to a lower extent, helping explain why the adaptation choice bias was weaker in the control experiment. Additional mechanisms during risk-averse agent predictions (i.e., response caution) were also present, suggesting that these predictions were not carried out via the exact same process as decisions for the self.

## Discussion

Our study aimed to provide experimental evidence that people use their own decision-making system when predicting the decisions of others (i.e., the simulation hypothesis). To do so, we first successfully biased participants’ valuation of risky options and then asked them to predict the decisions of distinct, hypothetical agents. We reasoned that if participants used their own decision-making system to make predictions for another person, a corresponding bias should be identified in the predicted decisions. Our results partly supported this hypothesis—on average, there was a difference in predicted acceptance rates between the two adaptation groups. Importantly, however, this difference was only present during prediction for the average and risk-averse, but not the risk-seeking agent. Also, we found that participants’ own risk tendencies correlated with predictions for the average and risk-averse, but not risk-seeking agent. Corroborated by mechanistic differences identified by the computational model, these findings suggest that people do employ simulations when predicting the decisions of another agent, but that they may only engage their own decision mechanisms when predicting the decisions of agents with certain characteristics.

Our findings generally support the view that people employ their own decision-making mechanism when predicting the decisions of someone else. As reviewed in the introduction, multiple strands of evidence point toward an overlap in mechanisms engaged during decision-making for the self and predicting the decisions of others.^13^^,^^16^^,^^17^^,^^18^^,^^28^ However, to our knowledge, our study is the first to directly show the involvement of participants’ decision-making process during prediction through experimental manipulation and, as such, provides additional support for the simulation hypothesis. As evidenced by the existence of ToM accounts which include both simulations and theories,^2^ findings consistent with simulation theory do not indicate that participants could not have used additional processes (for example, using general knowledge when deciding *how strongly* risk-seeking or risk-averse the other agent ought to be). Likewise, because our adaptation manipulation relies on a between-group aggregate effect, it is possible that some individuals may have relied on simulations when predicting for a particular agent, whereas others have not. Nevertheless, our results demonstrate that there is at least group-level reliance on one’s own cognitive system when making predictions for certain others.

A prominent and somewhat unexpected finding was that the above held true for predictions of the risk-averse and risk-average, but not the risk-seeking agent. In other words, participants’ prediction behavior was consistent with the simulation hypothesis (i.e., biased predictions, correlation of own and predicted risk tendencies) when making predictions for the risk-averse agent, whereas no such evidence was apparent during predictions for the risk-seeking agent. Such differences cannot be explained by information asymmetry across the stages^32^ since the same information was available across all stages and no feedback on participants’ predictions was given. Similarly, due to predictions not being incentivized, these results cannot be accounted for by participants assuming distinct reward biases or differences in stakes-weighing for the agents.^33^ More generally, because the task was to predict the decisions of hypothetical others, rather than choosing for concrete individuals, other factors such as self-image preservation or accountability were unlikely to differentially affect predictions for the various agents.^34^^,^^35^ Although we did not anticipate this when formulating the hypotheses, there is precedent for the idea that simulations may or may not be adopted, based on who is the target of inference or prediction. For instance, people seem to rely on simulation more when mentalizing for similar, rather than dissimilar agents.^36^^,^^37^^,^^38^ Our exploratory analysis did not, however, strongly support the notion that the more risk-averse half of participants showed a stronger correlation with the risk-averse agent predictions than the more risk-seeking half did (Appendix D). As such, it is conceivable that additional factors also informed the different prediction mechanism for the risk-seeking agent, such as the general difficulty in imagining or relating to the behavioral profile described in the vignette. Other characteristics, such as familiarity or closeness with the other agent, also affect the way predictions for others are made across various task domains.^39^^,^^40^^,^^41^^,^^42^^,^^43^^,^^44^ Future work could thus benefit from measuring or experimentally manipulating such factors to test whether they mediate the extent of reliance on one’s own decision system when making predictions.

A curious pattern worth addressing is that, in our study, the valuation bias in the control experiment was short-lived, whereas it persisted throughout the whole stage during the risk-averse agent predictions. The stability of this effect is not surprising by itself—the magnitude of the risk valuation bias in Guo & Tymula^25^ did not change over the time-course of the post-adaptation stage (60 trials of accepting/rejecting risky gambles for oneself), meaning that the stable group difference during prediction in our experiment could have plausibly arisen from the adaptation manipulation (but see Khaw et al.^24^). The gradual extinction of the bias in the control experiment and experiment 1 may point toward a potential mechanistic explanation: When making decisions for oneself/a weakly specified other, participants in our study may have gradually adapted to the range of probabilities within the post-adaptation option sets to evaluate individual decision options.^23^^,^^45^ This gradual range adaptation may have overwritten the relatively weak initial valuation bias induced by our manipulation. On the other hand, preferences of another person clearly distinct from oneself may be largely determined at the beginning of the decision period,^12^ meaning these assumed preferences are defined early and may be immune to subsequent context effects. Future work could leverage more sophisticated extensions of computational models that take into account the previous history of choice and contexts and include a dynamic, trial-to-trial valuation parameter to elucidate whether such an explanation holds. Nevertheless, this pattern (corroborated by the modeling results discussed in the next paragraph) indicates that, despite a clear valuation mechanism overlap, even the risk-averse agent’s predictions were not implemented as a “pure” simulation of one’s own decision process, and further work is required to identify the nuanced differences.

Comparing different evidence accumulation models revealed that, across conditions, participants did accumulate evidence based on the option values and that predictions were not performed via a probability matching heuristic strategy. It has also revealed that, in all conditions where the post-adaptation effect was present, a corresponding difference in the α valuation parameter was observed. This interpretation aligns with the simulation hypothesis, since it demonstrates that participant’s option valuation remained biased even during predictions and, conversely, that it remained unaffected in the risk-seeking other predictions, where a behavioral adaptation effect was absent. As recent accounts suggest,^46^ this may possibly reflect a bias in the lower-level process of encoding the numeric values of option magnitudes. Interestingly, in choices for the self, the adaptation manipulation also increased the predisposition to accept the option that was chosen more frequently in the adaptation stage (via the starting point bias parameter), suggesting that decision inertia was induced on top of the valuation bias.^47^ Such difference was smaller in predictions for all agents, indicating that individuals only partly carry over these response tendencies when predicting others. The fact that the manipulation also affected response caution during predictions for the risk-averse agent further indicates that, although valuation mechanisms are common to decisions for self and predictions alike, the implementation of predictions may differ from the process underlying decisions for self. Further work could build upon these findings and investigate how the parameters change across self-decisions and predictions, focusing on within-subjects dynamics (rather than focusing on differences between subjects as done here). Thereto, models with more complex and dynamic architectures will be required to determine precisely how people shift between their own and others’ preferences over time (e.g., in a gradual “anchoring-and-adjustment” fashion^48^^,^^49^).

Another important question for future research is whether our results extrapolate to different prediction situations. For instance, the phrasing of the instructions matters; asking participants to choose on behalf of the other person, to predict what the person would choose, or to predict what the person should choose can lead to different choice behavior.^26^^,^^50^ The instructions in our task simply asked the participants to “choose the way they would expect the other person to choose” in the prediction part, leaving the possibility that not all participants interpreted the task in the same way. The agents in the current study were also hypothetical, meaning that participants had to construct the other person’s risk preferences from a purely verbal description. Another common method for studying predicted decisions is to make participants learn the other agent’s preference by observing their choices,^13^^,^^15^^,^^51^ potentially making the target more vivid and concrete, which can change the way predictions are made.^52^ In the real world, our knowledge about the preferences of a person whose decision we predict often consists of vague and anecdotal information (e.g., that they like to save money; that they generally prefer fruits to sweets and so forth^53^)—not unlike the vignette descriptions utilized herein or in other studies.^54^ However, decision literature has identified a robust “description-experience gap” whereby decisions under uncertainty differ when the information is delivered via description, compared to when it is experienced via samples or observation.^55^^,^^56^ In the future, it would be interesting to examine whether our core findings extend to contexts where richer information about the other agent and their preferences (or at least feedback about the correctness of the predictions) is available, and whether it promotes simulation or, conversely, whether the more precise knowledge about the other’s preference makes the need for simulation redundant. In a similar effort to test the generality of the findings, future studies would benefit from utilizing loss-only or mixed-gambles (rather than gains-only options used throughout the present study), since in some studies the patterns in decisions for others differ across gain and loss contexts.^57^

In summary, our study contributed to the literature on interpersonal decision-making and ToM by experimentally demonstrating that predicted choices for others depend on the same cognitive system used to make decisions for oneself. Crucially, this is the case only when predicting for others with certain characteristics.

### Limitations of the study

The study has used a between-subjects manipulation—assigning participants to one of the two decision contexts—to induce a bias in their decision process. Additionally, no baseline, unbiased measure of participants’ decision behavior was collected before this adaptation phase. These factors, combined with the post-adaptation bias being assessed across three experiments with different participant samples, make it difficult to establish how the decision behavior and the adaptation bias change across the different decision and prediction conditions on an individual level. Another limitation of the study is that participants were not asked to provide subjective ratings about the hypothetical agents (e.g., similarity, familiarity, difficulty of predicting). Without such data and without an objectively “correct” way of making predictions, it can only be speculated how participants interpreted the verbal description of the agents and why they re-used their valuation mechanisms during prediction for some, but not all the agents.

## Resource availability

### Lead contact

Requests for further information and resources should be directed to and will be fulfilled by the lead contact, Erik Stuchlý (erik.stuchly@uni-hamburg.de).

### Materials availability

This study did not generate new unique reagents.

### Data and code availability

The behavioral data and code used for all analyses in this study are available at Open Science Framework: https://osf.io/svyma/. Any additional information required to reanalyze the data reported in this paper is available from the lead contact upon request.

## Acknowledgments

This work was supported by the 10.13039/501100000781European Research Council (ERC) under the European Union’s Horizon 2020 Research and Innovation Program (grant agreement no. 948545), awarded to Sebastian Gluth.

## Author contributions

E.S.: conceptualization, methodology, software, formal analysis, investigation, writing – original draft, and visualization. S.B.: conceptualization, methodology, validation, and writing – review and editing. S.G.: conceptualization, methodology, writing – review and editing, supervision, and funding acquisition.

## Declaration of interests

The authors declare no competing interests.

## STAR★Methods

### Key resources table

**Table:** 

REAGENT or RESOURCE	SOURCE	IDENTIFIER
**Deposited data**		

Behavioral data of participants	This paper	Open Science Framework: https://osf.io/svyma/files/osfstorage

**Software and algorithms**		

OpenSesame	Mathôt et al.^58^	https://osdoc.cogsci.nl/
R v4.4.2	R Core Team^59^	https://www.r-project.org/
RStudio 2023.06.0	RStudio Team^60^	https://posit.co/download/rstudio-desktop/
rstan	Stan Development Team^61^	https://mc-stan.org/rstan/

### Experimental model and study participant details

The following pre-registered methods have been approved by the Local Ethics Commitee of the Faculty for Psychology and Movement Sciences at the University of Hamburg (approval code 2022_019).

Data from 266 participants (Control experiment), 266 participants (Experiment 1), and 199 participants (Experiment 2) were included in the analyses. These sample sizes were determined by two power analyses computed in G∗power,^62^ designed to identify the respective between-group effect in the post-adaptation stages in each experiment. For the Control experiment and Experiment 1, a power analysis for a two-tailed, between-subjects *t* test, assuming the power of 0.9 and α of 0.05, indicated that in order to identify an expected effect with the Cohen’s d size of 0.4 (moderate), a sample of 266 participants should be obtained. For Experiment 2, a power analysis for a between-subjects (2 probability groups), repeated measures (2 prediction stages) ANOVA, assuming the power of 0.9 and α of 0.05, indicated that in order to identify an expected effect with the F size of 0.2 (moderate), 200 participants should be recruited. Participants with incomplete datasets or who failed the attention check were excluded, and another participant was recruited in their place. This was the case for 40 individuals in the Control experiment (6 incomplete datasets/technical issues, 34 failed attention checks), for 64 individuals in Experiment 1 (25 incomplete datasets/technical issues, 38 failed attention checks), and for 62 individuals in Experiment 2 (37 incomplete datasets/technical issues, 25 failed attention checks).

Participants were recruited via the online platform Prolific and as such, the sample’s demographics were expected to be representative of an online sample, based predominantly in the United Kingdom. The mean age was 31.6 (sd= 9.8) years with 107 female/158 male in the Control experiment; 33.3 (sd= 11.1) years with 91 female/173 male/1 not specified in Experiment 1; and 35.6 (sd= 11.7) years with 87 female/109 male/3 not specified in Experiment 2. Potential participants were required to be at least 18 years old, have normal/corrected to normal vision and access to a computer with a keyboard, and they must not have participated in any of the other two experiments. Additionally, participants must have provided informed consent before starting the experimental task.

Participants were financially compensated with a fixed base payment of 6 EUR (8 EUR in Experiment 2) for successfully completing the whole experiment, with the potential to earn an additional bonus payment. The value of this bonus was based on the outcome of a randomly chosen gamble that participant selected during the experiment, which was on average 1.27 EUR.

### Method details

The study employed a mixed design; in the Control experiment and Experiment 1, we used a 2×2 design (Figure 1), with stage (adaptation and post-adaptation) as a within-subjects factor and probability group in the adaptation stage (high probability vs. low probability) as a between-subjects factor. In Experiment 2, we used a 3×2 design (Figure 1C), with stage (adaptation - self, prediction - risk-averse, prediction - risk-seeking) as the within-subjects factor and probability group in the adaptation stage (high probability vs. low probability) as the between-subjects factor.

A risky choice paradigm was utilized throughout all stages. In each trial, participants had to choose between a safe option - winning a smaller number of points for sure - or a risky option - a certain probability of obtaining a higher number of points, otherwise gaining nothing (for more details on how the option values were generated, see the Option generation subsection). No losses were possible in the experiment, as both the safe and risky options were associated with a possible gain (or no gain in case of the risky option). The option set was identical across participants, but the individual option pairs within a particular stage were presented in a fully randomized order for each participant.

At the beginning of the experiment, participants read and signed the electronic consent sheets, read task instructions and then completed 12 practice trials (similar but not identical to the trials encountered in later stages) to familiarize themselves with the risky choice task, which looked as follows (Figure 1B): A fixation cross appeared at the center of the screen for 1 s, after which the safe and risky option were presented on the left and right side of the screen, with an annotated piechart representing the probability of winning the option and a number indicating the possible gains (number of points) for each option. The position of the safe and risky options on the computer screen were counterbalanced across trials (the safe option appeared on the left side in half of the trials). The options had been presented on the screen until a response was made with the “f” or “j” key, at which point the chosen option was highlighted with a white rectangle for 1.5 s, followed by feedback about the outcome of the trial, displayed for 1.5 s. If the participant did not make a choice within 10 s, they were informed that they were too slow (displayed for 3 s) and that they did not get any points (<1 timed-out trials per participant on average; these trials were not included in the analyses). Afterward, the next trial started.

At the beginning of the adaptation stage, participants were instructed to make risky decisions for themselves and were informed that one of their choices would be randomly selected at the end of the experiment as a bonus payment. Importantly, they were randomly allocated to one of two groups (unbeknownst to them), both of which encountered decision sets with the same safe option outcomes, but the attractiveness of the risky option was manipulated to bias participant’s risk valuation.^25^ Specifically, half the participants were assigned to a “low probability” group (LP), where the risky option had a relatively low average probability of payout (Figure 1A). The other half were allocated to the “high probability” group (HP), where the risky option had a higher average probability of payout. The mean value difference between the safe and risky option were specifically selected for each group (see Table 1 in the Option generation subsection), such that the LP group would encounter mostly unappealing risky options on average, whereas the HP group would encounter mostly attractive risky options. Altogether, participants completed 104 decisions in this stage, with one self-paced break after 1/3 and 2/3 of trials each.

The “post-adaptation/prediction” stage was conceptually similar, with a few key differences. In the Control experiment, participants were simply instructed to keep making decisions for themselves. In Experiment 1, the instruction was to imagine an “average, typical person” and to decide the way they would expect them to decide. In Experiment 2, this stage began with a presentation of a vignette describing a hypothetical agent behaving in a very risk-seeking or very risk-averse manner across multiple behavioral domains:

You will now be making decisions on behalf of another person, for whom the following applies.

• They would bet a day’s income on the outcome of a sporting event.
• When driving, they frequently go over the speed limit and overtake other cars.
• They usually download movies from illegal websites.
• They invest 5% of their annual income into highly volatile stocks.

You will now be making decisions on behalf of another person, for whom the following applies.

• They would avoid betting a day’s income on the outcome of sports events.
• When driving, they stick to the speed limit and do not overtake unless they absolutely have to.
• They usually stream their movies on legal, secure platforms.
• They put 5% of their annual income into a savings account.

The statements were adapted from the DOSPERT questionnaire,^63^ chosen to represent various risk domains (i.e., financial-gambling, health, ethical, financial-investing) and phrased as similarly as possible across the two vignettes. Notably, similar vignettes have been successfully used to describe a hypothetical decision-maker with a cautious or careless decision style in Gates et al.^54^ Subsequently, participants were asked to choose the way they would expect the described agent to choose in this stage. All participants in Experiment 2 completed the prediction stage twice, once for each agent in a block-wise fashion, with the order of agent presentation counterbalanced across participants. The second key difference in the post-adaptation stage (across all three experiments) was that all participants saw the same option set, regardless of whether they were previously allocated to the HP/LP group. Specifically, the probability range in this stage was higher/lower than in the LP/HP adaptation stage, respectively, as was the mean value difference of the option set (please refer to the Option generation subsection for more detail). This stage consisted of 100 trials per agent, again with the opportunity to take a break after 1/3 and 2/3 of the trials.

In Experiment 1 only, after the post-adaptation stage, participants were presented with a slider scale from 0 to 100 and asked to indicate their own risk preference (from “very risk-seeking” to “very risk-averse”), and to indicate how different from themselves (from “much more risk-seeking” to “much more risk-averse”) they imagined the other person to be. At the end of all experiments, participants were informed about their bonus payment and the experiment ended.

To ensure data quality, an attention check was employed in all three experiments. The adaptation stage in all three experiments included four “catch” trials - that is, trials where the probability of winning the risky gamble was either 100% (HP group) or 0% (LP group). Provided that participants were engaged with the task, they should have always accepted the risky option in the HP group, whereas they should have rejected it in the LP group. If they responded incorrectly at least 1 out of the 4 times, their data was excluded and another participant was recruited in their place. In the prediction stage of Experiment 2, four additional comprehension questions (i.e., two questions when predicting for the risk-seeking agent, two questions when predicting for the risk-averse agent) were asked, where participants had to indicate which of the two agents they were currently making predictions for. In total, Experiment 2 contained 8 awareness checks – 4 catch trials and 4 questions about the other agent. If the participant responded incorrectly more than 1 out of 8 times, their data was excluded and another participant was recruited in their place. Altogether, participants in the control experiment and Experiment 1 completed 204 trials (approx. 35 min), and participants in Experiment 2 completed 304 trials (approx. 50 min). The entire task was coded in OpenSesame^58^ and hosted on JATOS online servers.^64^

#### Option generation

We used value differences as computed by Prospect Theory^65^ to create an option set where participants in the adaptation stage would accept the risky option about 75% of the time in the HP group, and about 25% of the time in the LP group. The subjective value difference between the safe and risky option on any given trial is defined as:

(Equation 1) $$VD=v_{\mathrm{risky}}*w_{\mathrm{risky}}-v_{\mathrm{safe}}*w_{\mathrm{safe}}$$

where

(Equation 2) $$v_{o}=x_{o}^{\alpha }$$

(Equation 3) $$w_{o}=\frac{p_{o}^{\gamma }}{{\left(p_{o}^{\gamma }+{\left(1-p_{o}\right)}^{\gamma }\right)}^{\frac{1}{\gamma }}}$$

where x denotes the potential monetary outcome of option o and p denotes the probability of obtaining the outcome. α represents a “riskiness” parameter which governs how strongly monetary values are discounted, and the parameter γ governs the distortion of perceived probabilities. To generate decision options that, according to Equation 1, had a positive VD more often in the HP group, and a negative VD more often in the LP group, we rearranged said equation into

(Equation 4) $$x_{\mathrm{risky}}=\sqrt[{\alpha }]{\frac{x_{\mathrm{safe}}^{\alpha }*1-VD}{\frac{p_{\mathrm{risky}}^{\gamma }}{{\left(p_{\mathrm{risky}}^{\gamma }+{\left(1-p_{\mathrm{risky}}\right)}^{\gamma }\right)}^{\frac{1}{\gamma }}}}}$$

For the α and γ parameter values, we took the averages commonly reported in the risky choice literature (α=0.8 and γ=0.8).^66^ In terms of behavior, these values translate into mildly risk-averse behavior, with slight underweighting of medium-range probabilities and exaggerated perception of extreme probabilities. Using all combinations of 2 safe option outcomes (15 and 25 points) and 6 risky option probabilities allowed us to generate 12 unique option pairs with the same VD. We repeated this procedure for 5 VD levels distinct for each experimental stage (as listed in Table 1). Furthermore, to make the option sets more easily discriminable for the participants, we have used different values of risky option probabilities across stages (displayed in Figure 1A and listed in the pre-registration protocol), such that the option sets with lower average VD were also associated with lower average risky option probabilities. Thus, we created three option sets with 50 unique decision options each, where participants would favor more risky options on average in the Adaptation HP set, fewer risky options in the Adaptation LP set, and a roughly equal proportion of safe and risky options in the post-adaptation/prediction option set. Each of the 50 option pairs appeared twice within the respective option set.

### Quantification and statistical analysis

#### Behavioral data analysis

Behavioral analyses were conducted in R v4.4.2,^59^ using RStudio 2023.06.0.^60^ Comparison of means for the choice data in the post-adaptation stages were conducted with a paired samples *t* test for the Control experiment and Experiment 1, and with mixed ANOVA (Greenhouse–Geisser correction on within-subject factors), with probability group as the between-subjects factor and stage as the within-subjects factor for Experiment 2. Post-hoc tests were conducted with Bonferroni correction. Due to heteroscedasticity in the participant-level mean choice data, reported correlations between choice behavior in the different experimental stages were conducted with the Spearman method. Within all figures, the following conventions for depicting statistical significance are adopted: nsp>.10; †p<.10; ∗p<.05; ∗∗p<.01; ∗∗∗p<.001.

#### Computational modeling

To better understand the underlying dynamics of predicted decisions, we fitted the drift-diffusion model (DDM)^30^ to the data. Each of the model’s core parameters has a psychological interpretation - the drift rate represents a within-trial decision difficulty, largely governed by the stimulus/decision options; the threshold indicates response caution set by the decision-maker; the starting point represents the decision-maker’s initial bias for accepting one type of option over another, independent of the current evidence; and finally, nondecision time denotes the time taken for processes unrelated to accumulating evidence (such as visual encoding and response execution). Here we formulate an extension of the standard DDM, so that it can be applied to risky decisions.^67^^,^^68^^,^^69^^,^^70^^,^^71^ Specifically, we adopt the common assumption that on a value-based decision task, the value difference between the two options governs the speed and direction of evidence accumulation.^72^ In other words, drift rate on a particular trial should be driven by the subjective utility difference between the safe and the risky option:

(Equation 5) $$drift=\left({\left(v_{\mathrm{risky}}*w_{\mathrm{risky}}\right)}^{1/\alpha }-{\left(v_{\mathrm{safe}}\right)}^{1/\alpha }\right)*ds$$

Where *ds* is a drift scaling parameter which scales the difference into an appropriate range. $v_{\mathrm{option}}$ is the value function which transforms the objective outcome of an option into a subjective value from Equation 2, with the α parameter determining the degree to which monetary outcomes get transformed (i.e., a “riskiness” parameter). $w_{\mathrm{risky}}$ denotes the subjective probability of winning the gamble, as computed by Equation 3. To de-correlate the values of α and *ds* parameters, we have raised the subjective value of the safe and risky option to 1/α (see Steward et al.^73^ for more detail).

As such, the full model (which we will refer to as “Prospect Theory Drift Diffusion Model” - PT-DDM) consisted of six parameters: α, γ, drift scaling (ds), threshold (τ), nondecision time (ndt) and starting point bias (sp). We compared goodness-of-fit of this model against three simpler version of the model, the first of which assumes that the drift rate is driven by the utility difference between the safe and risky option, but without any distortion in the probability representation (γ = 1). As such, this model specification (which we refer to as “Expected Utility Drift Diffusion Model” - EU-DDM) consisted of 5 free parameters: α, drift scaling (ds), threshold (τ), nondecision time (ndt) and starting point bias (sp). The second alternative model assumes that the drift rate is driven by the expected value difference between the safe and risky option, reflecting the assumption that participants do not convert the expected values of the options into subjective utilities when making their decision (α = 1, γ = 1). As such, this model specification (which we refer to as “Expected Value Drift Diffusion Model” - EV-DDM) only consisted of 4 parameters: drift scaling (ds), threshold (τ), nondecision time (ndt) and starting point bias (sp). The last model assumed that participants ignored trial-level information, and instead aimed for a fixed proportion of accepted risky options within a stage (similar to probability matching^74^). This model (which we refer to as “Heuristic Drift Diffusion Model” - heur-DDM) also consisted of four parameters - drift rate (ds), threshold (τ), nondecision time (ndt) and starting point bias (sp). Unlike in the previous models though, the drift rate of this model was not modulated by option-related information.

The models were implemented in the probabilistic Stan language, interfacing with RStudio through the rstan package.^61^^,^^75^ The posterior distributions of parameters were obtained via sampling with the No-U-Turn Sampler,^76^ with 4 parallel chains ran over 2000 iterations, the first 750 of which served as warm-up. We fitted the models to data from all conditions and groups of each experiment jointly (4 sub-groups in Control experiment and Experiment 1, 6 sub-groups in Experiment 2) using a hierarchical Bayesian approach, meaning that both group-level parameters for each group/experimental condition and individual-level parameters for each participant within were jointly estimated and informed each other (for more details on model parameterization and parameter priors, please refer to Appendix F).

The four models were compared using the approximation of the leave-one-out cross-validation (LOO),^77^ which is based on each model’s expected log predictive densities (ELPD) as a measure of out-of-sample predictive accuracy. In our instantiation, the LOO method compares the ELPD of the models by leaving out datasets of a single participant and computing the metric on the rest of the dataset. Having obtained the ELPD values of each model, it is possible to compare the relative performance of the model by taking the models’ difference on this measure and express them in a LOOIC metric (lower values indicating a better model fit). Following the standard convention, a model was assumed to provide a meaningfully worse fit than the best one if the magnitude of the ELPD difference was > 2 times higher than the standard error of the ELPD difference between the two models. In addition, model weights were computed via pseudo-BMA+ with Bayesian bootstrap, as well as participant win counts.

To further validate the best-fitting model and check whether the fitted parameter values are able to generate data similar to real participants’ responses, we conducted a posterior predictive check for each experimental sub-condition (combination of probability group and agent/stage). We simulated the full dataset of each condition 500 times, each of which was simulated with parameters from the best-fitting iteration in the posterior parameter space (i.e., highest negative log likelihood). This effectively gave us 500 means of choice and RTs (separately for safe and risky choices); we then computed the 95% highest density interval (HDI) of the simulated mean distribution for each condition and compared the mean choice/RT in the real data against this HDI.

To identify mechanistic differences between experimental groups, we extracted the posterior distribution draws for each parameter from the fitted model, computed the difference of each draw between the HP and LP groups in the corresponding post-adaptation condition and, finally, took the 95% HDI of this difference (i.e., the lower and upper bound that contain 95% of the resulting probability distribution’s density). We refer to parameter values as “credibly different” across the two groups if zero falls outside the bounds of this HDI (although we emphasize that it does not represent a categorical boundary between “statistically significant” and “statistically not significant” difference).

### Additional resources

The recruitment methods, design and procedure, and the data analysis plan were pre-registered on the Open Science Framework Website (https://osf.io/svyma/?view_only=8b4c13df5f3c4fbe9d2a46d33c57dfbd). The following three hypotheses related to the research question were also pre-registered.

1. Biasing participants’ own risk preference when they decide for themselves will result in a corresponding bias when they predict decisions on behalf of another agent.
2. We predict that higher value difference (VD) and overall value (OV) of the options will lead to lower RTs, both in decisions for oneself and in predicted decisions.
3. The participant-level measure of risk attitude should correlate between the decision and prediction stages.

We note that hypothesis 2 is evaluated in Appendix E, due to the fact that our OV analyses of decisions for oneself yielded results inconsistent with established literature, thus decreasing our confidence in the observed patterns and their suitability for comparison with predicted decisions. Additionally, the generalised linear models as applied to choice data were instead replaced with t-tests/ANOVAs in the manuscript, since they provided a more appropriate and interpretable test of hypothesis 1. The results from the pre-registered linear models can be seen in Tables S3 and S4 in Appendix E. The code and data analyzed in this study can be freely accessed at the following link: https://osf.io/svyma/files.

## References

[1] Frith C.D., Frith U. (2006). The neural basis of mentalizing. Neuron.

[2] Davies M., Stone T. (1998). Folk Psychology and Mental Simulation. Roy. Inst. Philos. Suppl..

[3] Saxe R. (2005). Against simulation: The argument from error. Trends Cognit. Sci..

[4] Bazinger C., Kühberger A. (2012). Is social projection based on simulation or theory? why new methods are needed for differentiating. New Ideas Psychol..

[5] Baker C.L., Saxe R., Tenenbaum J.B. (2009). Action understanding as inverse planning [Number: 3]. Cognition.

[6] Nguyen T.N., Gonzalez C. (2022). Theory of mind from observation in cognitive models and humans. Top. Cogn. Sci..

[7] Milosavljevic M., Malmaud J., Huth A., Koch C., Rangel A. (2010). The drift diffusion model can account for the accuracy and reaction time of value-based choices under high and low time pressure. Judgm. Decis. Mak..

[8] Busemeyer J.R., Gluth S., Rieskamp J., Turner B.M. (2019). Cognitive and neural bases of multi-attribute, multi-alternative, value-based decisions. Trends Cognit. Sci..

[9] Polanía R., Krajbich I., Grueschow M., Ruff C.C. (2014). Neural oscillations and synchronization differentially support evidence accumulation in perceptual and value-based decision making. Neuron.

[10] Gluth S., Rieskamp J., Büchel C. (2012). Deciding when to decide: Time-variant sequential sampling models explain the emergence of value-based decisions in the human brain. J. Neurosci..

[11] Tarantola T., Kumaran D., Dayan P., De Martino B. (2017). Prior preferences beneficially influence social and non-social learning. Nat. Commun..

[12] Harris A., Clithero J.A., Hutcherson C.A. (2018). Accounting for taste: A multi-attribute neurocomputational model explains the neural dynamics of choices for self and others. J. Neurosci..

[13] Smith S.M., Krajbich I. (2023). Predictions and choices for others: Some insights into how and why they differ. J. Exp. Psychol. Gen..

[14] Devaine M., Daunizeau J. (2017). Learning about and from others’ prudence, impatience or laziness: The computational bases of attitude alignment. PLoS Comput. Biol..

[15] Bavard S., Stuchlý E., Konovalov A., Gluth S. (2024). Humans can infer social preferences from decision speed alone. PLoS Biol..

[16] Nicolle A., Klein-Flügge M.C., Hunt L.T., Vlaev I., Dolan R.J., Behrens T.J. (2012). An agent independent axis for executed and modeled choice in medial prefrontal cortex. Neuron.

[17] Suzuki S., Harasawa N., Ueno K., Gardner J.L., Ichinohe N., Haruno M., Cheng K., Nakahara H. (2012). Learning to simulate others’ decisions. Neuron.

[18] Piva M., Velnoskey K., Jia R., Nair A., Levy I., Chang S.W. (2019). The dorsomedial prefrontal cortex computes task-invariant relative subjective value for self and other. eLife.

[19] Zhao W.J., Walasek L., Bhatia S. (2020). Psychological mechanisms of loss aversion: A drift-diffusion decomposition. Cogn. Psychol..

[20] Juechems K., Altun T., Hira R., Jarvstad A. (2022). Human value learning and representation reflect rational adaptation to task demands. Nat. Hum. Behav..

[21] Stewart N., Chater N., Stott H.P., Reimers S. (2003). Prospect relativity: How choice options influence decision under risk. J. Exp. Psychol. Gen..

[22] Spektor M.S., Bhatia S., Gluth S. (2021). The elusiveness of context effects in decision making. Trends Cognit. Sci..

[23] Bavard S., Rustichini A., Palminteri S. (2021). Two sides of the same coin: Beneficial and detrimental consequences of range adaptation in human reinforcement learning. Sci. Adv..

[24] Khaw M.W., Glimcher P.W., Louie K. (2017). Normalized value coding explains dynamic adaptation in the human valuation process. Proc. Natl. Acad. Sci. USA.

[25] Guo J., Tymula A. (2021). Waterfall illusion in risky choice – exposure to outcome-irrelevant gambles affects subsequent valuation of risky gambles. Eur. Econ. Rev..

[26] Macaskill A.C. (2021). Recently encountered delay lengths produce contrast effects on delay discounting. Behav. Processes.

[27] Bavard S., Lebreton M., Khamassi M., Coricelli G., Palminteri S. (2018). Reference-point centering and range-adaptation enhance human reinforcement learning at the cost of irrational preferences. Nat. Commun..

[28] Kühberger A., Luger-Bazinger C. (2016). Predicting framed decisions: Simulation or theory?. Psychology.

[29] Voss A., Rothermund K., Voss J. (2004). Interpreting the parameters of the diffusion model: An empirical validation. Mem. Cognit..

[30] Ratcliff R. (1978). A theory of memory retrieval. Psychol. Rev..

[31] Desai N., Krajbich I. (2022). Decomposing preferences into predispositions and evaluations. J. Exp. Psychol. Gen..

[32] Liu Y., Polman E., Liu Y., Jiao J. (2018). Choosing for others and its relation to information search. Organ. Behav. Hum. Decis. Process..

[33] Contreras-Huerta L.S., Pisauro M.A., Küchenhoff S., Gekiere A., Le Heron C., Lockwood P.L., Apps M.A.J. (2024). A reward self-bias leads to more optimal foraging for ourselves than others. Sci. Rep..

[34] Liu P.J., Baskin E. (2021). The quality versus quantity trade-off: Why and when choices for self versus others differ. Pers. Soc. Psychol. Bull..

[35] Javed A., Onculer A. (2026). Self-other discrepancy: The role of decision transparency in risky choices. J. Bus. Res..

[36] Woo B.M., Mitchell J.P. (2020). Simulation: A strategy for mindreading similar but not dissimilar others?. J. Exp. Soc. Psychol..

[37] O’Brien E., Ellsworth P.C. (2012). More than skin deep: Visceral states are not projected onto dissimilar others. Psychol. Sci..

[38] Schneider M., Tamir D., Rubin-McGregor J. (2025). Simulation requires activation of self-knowledge to change self-concept. J. Exp. Psychol. Gen..

[39] Zhang X., Liu Y., Chen X., Shang X., Liu Y. (2017). Decisions for others are less risk-averse in the gain frame and less risk-seeking in the loss frame than decisions for the self. Front. Psychol..

[40] Ziegler F.V., Tunney R.J. (2012). Decisions for others become less impulsive the further away they are on the family tree. PLoS One.

[41] Batteux E., Ferguson E., Tunney R.J. (2017). Risk preferences in surrogate decision making. Exp. Psychol..

[42] Montinari N., Rancan M. (2018). Risk taking on behalf of others: The role of social distance. J. Risk Uncertain..

[43] Ruessmann J.K., Topolinski S. (2020). Economic decisions for others are more favorable for close than distant clients. Pers. Soc. Psychol. Bull..

[44] Kim H., Schnall S., Yi D.-J., White M.P. (2013). Social distance decreases responders’ sensitivity to fairness in the ultimatum game. Judgm. Decis. Mak..

[45] Rigoli F., Chew B., Dayan P., Dolan R.J. (2018). Learning contextual reward expectations for value adaptation. J. Cognit. Neurosci..

[46] Khaw M.W., Li Z., Woodford M. (2021). Cognitive imprecision and small-stakes risk aversion. Rev. Econ. Stud..

[47] Senftleben U., Schoemann M., Rudolf M., Scherbaum S. (2021). To stay or not to stay: The stability of choice perseveration in value-based decision making. Q. J. Exp. Psychol..

[48] Epley N., Keysar B., Van Boven L., Gilovich T. (2004). Perspective taking as egocentric anchoring and adjustment. Journal of Personality and Social Psychology.

[49] Wang Y.A., Simpson A.J., Todd A.R. (2023). Egocentric anchoring-and-adjustment underlies social inferences about known others varying in similarity and familiarity. J. Exp. Psychol. Gen..

[50] Tunney R.J., Ziegler F.V. (2015). Toward a psychology of surrogate decision making. Perspect. Psychol. Sci..

[51] Suzuki S., Jensen E.L.S., Bossaerts P., O’Doherty J.P. (2016). Behavioral contagion during learning about another agent’s risk-preferences acts on the neural representation of decision-risk. Proc. Natl. Acad. Sci. USA.

[52] Hsee C.K., Weber E.U. (1997). A fundamental prediction error: Self–others discrepancies in risk preference. J. Exp. Psychol. Gen..

[53] Jung M.H., Moon A., Nelson L.D. (2020). Overestimating the valuations and preferences of others [Place: US Publisher: American Psychological Association]. J. Exp. Psychol. Gen..

[54] Gates V., Callaway F., Ho M.K., Griffiths T.L. (2021). A rational model of people’s inferences about others’ preferences based on response times. Cognition.

[55] Wulff D.U., Mergenthaler-Canseco M., Hertwig R. (2018). A meta-analytic review of two modes of learning and the description-experience gap. Psychol. Bull..

[56] Hertwig R., Erev I. (2009). The description–experience gap in risky choice. Trends Cognit. Sci..

[57] Sun Q., Zhang H., Zhang J., Zhang X. (2018). Why can’t we accurately predict others’ decisions? prediction discrepancy in risky decision-making. Front. Psychol..

[58] Mathôt S., Schreij D., Theeuwes J. (2012). OpenSesame: An open-source, graphical experiment builder for the social sciences. Behav. Res. Methods.

[59] R Core Team (2020). https://www.R-project.org/.

[60] RStudio Team (2019). http://www.rstudio.com/.

[61] Stan Development Team (2025). RStan: The R interface to Stan [R package version 2.32.7]. https://mc-stan.org/.

[62] Faul F., Erdfelder E., Buchner A., Lang A.-G. (2009). Statistical power analyses using g∗power 3.1: Tests for correlation and regression analyses. Behav. Res. Methods.

[63] Blais A.-R., Weber E.U. (2006). A domain-specific risk-taking (DOSPERT) scale for adult populations. Judgm. Decis. Mak..

[64] Lange K., Kühn S., Filevich E. (2015). “just another tool for online studies” (JATOS): An easy solution for setup and management of web servers supporting online studies. PLoS One.

[65] Tversky A., Kahneman D. (1992). Advances in prospect theory: Cumulative representation of uncertainty. J. Risk Uncertain..

[66] Fox C.R., Poldrack R.A., Glimcher P.W., Camerer C.F., Fehr E., Poldrack R.A. (2009). Neuroeconomics.

[67] Sheng F., Ramakrishnan A., Seok D., Zhao W.J., Thelaus S., Cen P., Platt M.L. (2020). Decomposing loss aversion from gaze allocation and pupil dilation. Proc. Natl. Acad. Sci. USA.

[68] Konovalov A., Krajbich I. (2019). Revealed strength of preference: Inference from response times. Judgm. Decis. Mak..

[69] Peters J., D’Esposito M. (2020). The drift diffusion model as the choice rule in inter-temporal and risky choice: A case study in medial orbitofrontal cortex lesion patients and controls. PLoS Comput. Biol..

[70] Glickman M., Sharoni O., Levy D.J., Niebur E., Stuphorn V., Usher M. (2019). The formation of preference in risky choice. PLoS Comput. Biol..

[71] Diederich A., Trueblood J.S. (2018). A dynamic dual process model of risky decision making. Psychol. Rev..

[72] Ting C.-C., Gluth S. (2024). Unraveling information processes of decision-making with eye-tracking data. Front. Behav. Econ..

[73] Stewart N., Scheibehenne B., Pachur T. (2018). Psychological parameters have units: A bug fix for stochastic prospect theory and other decision models. PsyArXiv.

[74] Vulkan N. (2000). An economist’s perspective on probability matching. J. Econ. Surv..

[75] Carpenter B., Gelman A., Hoffman M.D., Lee D., Goodrich B., Betancourt M., Brubaker M., Guo J., Li P., Riddell A. (2017). Stan: A probabilistic programming language. J. Stat. Softw..

[76] Betancourt M. (2018). A conceptual introduction to hamiltonian monte carlo. arXiv.

[77] Vehtari A., Gelman A., Gabry J. (2017). Practical bayesian model evaluation using leave-one-out cross-validation and WAIC. Stat. Comput..

