# Supplementary material for: Deciding to simulate: Cognitive mechanisms of predicting the decisions of others
Source: iScience. 2026 Jun 24;29(7):116047. doi: 10.1016/j.isci.2026.116047 (PMC13319925; doi:10.1016/j.isci.2026.116047)
Supplement: Document S1. Figures S1–S7, and Tables S1–S8 [file mmc1.pdf]

iScience, Volume *29*

Supplemental information

Deciding to simulate: Cognitive mechanisms of predicting the decisions of others

Erik Stuchlý, Sophie Bavard, and Sebastian Gluth

Supplemental information

Appendix A - Response time data

Figure S1

Group-level response time data.

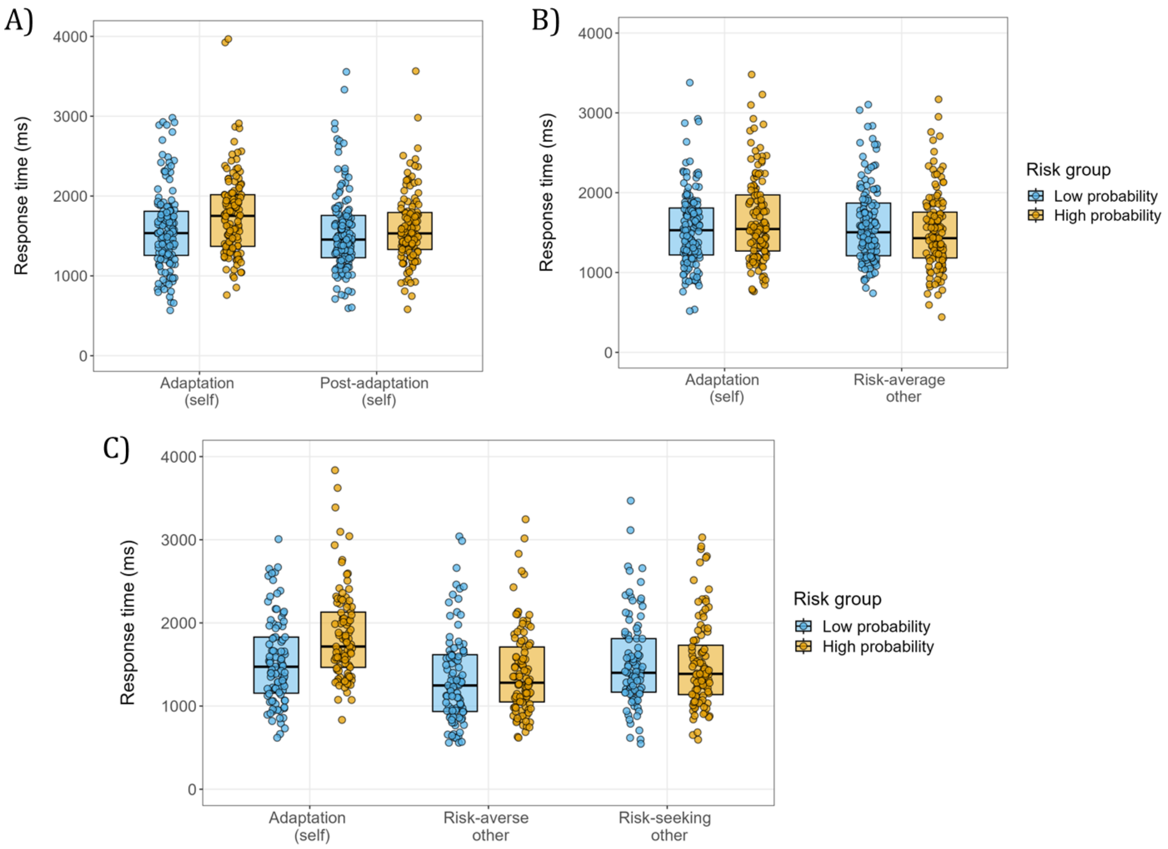

Note: Response time distributions from the Control experiment (A), Experiment 1 (B) and Experiment 2 (C). Individual points indicate the mean response time of a single participant, with the thick line denoting the group-level median and the upper/lower edge of the box representing the 25th/75th percentile of the distribution.

Within each experiment, we compared the response time distributions from each stage and risk group with a generalised linear model. Following the recommendations of Lo & Andrews[1], we Winsorised the data from each sub-group (0.05 and 0.95 percentiles) and then applied the following model with a Gamma distribution and identity link function:

$$RT \sim\ 1 + stage + risk\_group + stage * risk\_group$$

The main effects of stage were then assessed with post-hoc test implemented with the R package emmeans[2].

Appendix B - Accounting for the floor/ceiling effects in Experiment 2

Figure S2

Group-level data for trials with different value differences.

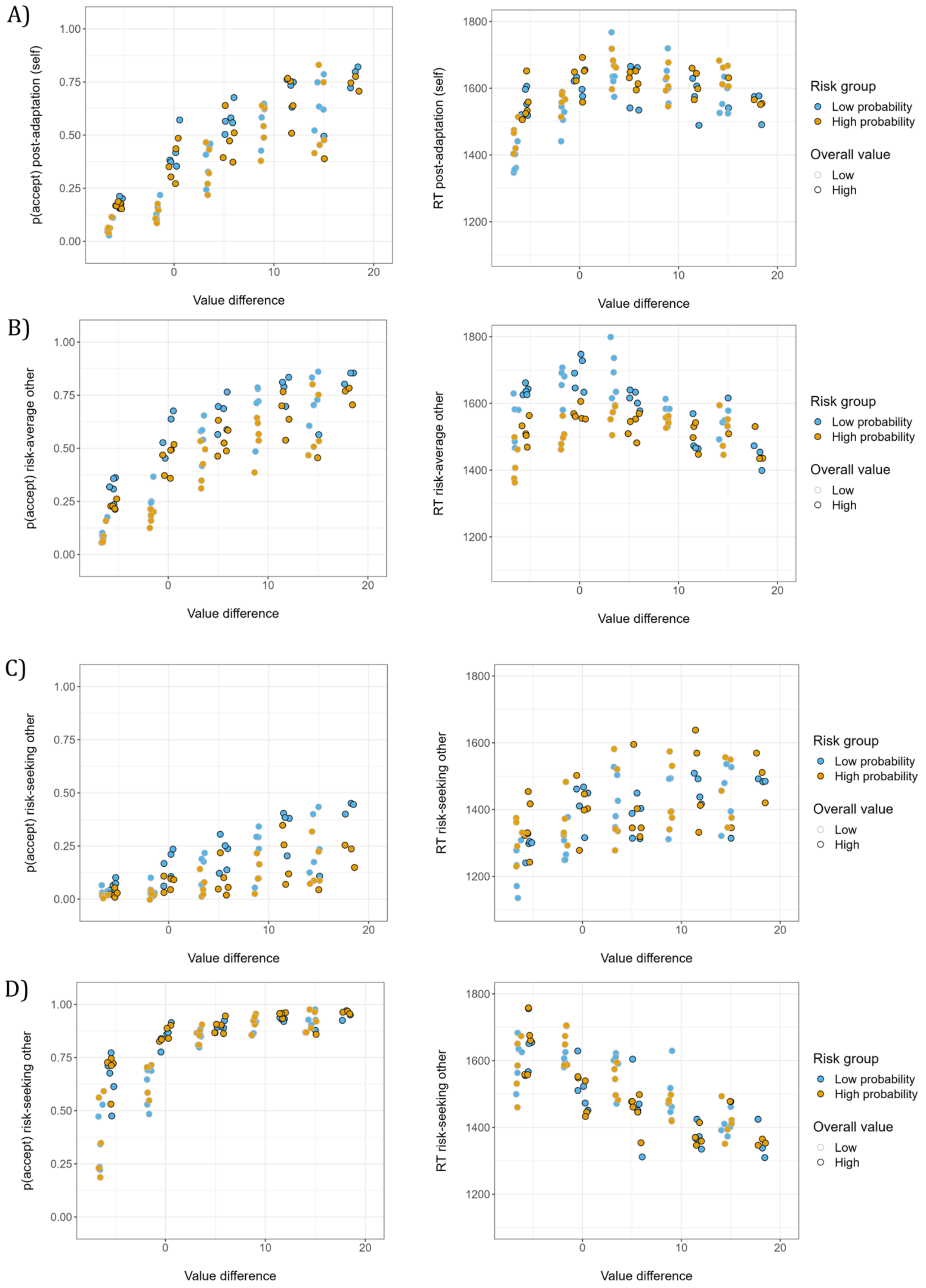

Note: Acceptance rates of the risky option (left) and the mean RTs (right) across trials with different expected value differences in the post-adaptation stages of all experiments: A) control experiment post-adaptation stage. B) Experiment 1, average other stage. C) Experiment 2, risk-averse other stage. D) Experiment 2, risk-seeking other stage. Individual points indicate the mean for a unique trial (i.e., unique combination of risky and safe option) across all participants, sub-divided into the HP and LP group, as well as by the overall value level (i.e., whether the magnitude of the safe option was 10 points or 25 points).

As Figure S2 (left) shows, many trials in the prediction stages of Experiment 2 were clustered at the ceiling/floor, which could possibly reduce chances of detecting a group effect in p_{accept} (especially for the risk-seeking agent, where no such effect was identified). To quantify the extent of ceiling and floor effects in the prediction stages of Experiment 2, we have used the rec.mean.var function in the DACF R package[3] to estimate how many participants performed at floor/ceiling levels. The proportion in the risk-seeking agent predictions was at 3%; in contrast, the proportion of datapoints in the risk-averse agent predictions reached 31%. Given that the adaptation effect was detected in the risk-averse prediction despite the much larger presence of floor effects than in the risk-seeking agent predictions, the relatively minor degree of ceiling effects in the latter stage could not be solely responsible for the lack of a group difference. To test this formally, we have used the t-test function offered by the same package (lw.t.test), which uses a truncated distribution assumption to compare group-level means. The difference has persisted in risk-averse prediction ($t = 4.428, d = 0.614, p < .001$), whereas no difference was apparent in risk-seeking predictions ($t = 0.458, d = 0.103, p = .458$), confirming the analysis pattern from the main text.

Appendix C - Assessing the change in choice behaviour during post-adaptation stages

To test whether the p_{accept} tendency initially increased/decreased and then plateaued over the post-adaptation/prediction stage, we fitted the choice data with an exponential decay model implemented in the brms package[4]:

$$choice \sim invlogit(\mathbf{a} + \mathbf{b} * e^{(-\lambda * trial)})$$

This non-linear model assumes that the choice proportion begins at a baseline (given by the a parameter) and that it shifts towards a new choice proportion level (parameter b) at a certain rate over trials (parameter λ). We fitted this model to data from each post-adaptation stage, separately for HP and LP groups and in the case of Experiment 2, separately for individuals who predicted the risk-averse agent first and those who predicted the risk-seeking agent first. To assess whether there was a shift in choice behaviour over time, we focused on the b parameter's posterior distribution: if the 95% HDI of this parameter included 0, it means there was no substantial shift from the baseline choice proportion over time. As Table S1 shows, the choice rate has credibly changed in Control experiment and Experiment 1, whereas the intervals from all conditions in Experiment 2 include 0 and thus indicate relative stability in choice behaviour across trials, confirming the qualitative patterns.

Table S1

Posterior group-level parameter HDIs of the exponential decay model in each post-adaptation stage.

	a		*b*		*λ*	
	LP	HP	LP	HP	LP	HP
Control experiment	[-9.300 -3.223]	[-9.173 -3.345]	[-8.291 -2.300]	[-8.325 -2.459]	[0.003 0.027]	[0.003 0.026]
Experiment 1	[-8.583 -4.413]	[-8.646 -4.386]	[-7.036 -2.688]	[-7.007 -2.701]	[0.011 0.042]	[0.011 0.042]
Experiment 2, RA (RA first)	[-9.241 -3.438]	[-8.986 -2.779]	[-5.363 3.624]	[-6.146 3.604]	[0.001 3.957]	[0.000 3.844]
Experiment 2, RS (RA first)	[-7.494 -2.040]	[-7.474 -2.076]	[-6.173 1.265]	[-5.965 1.608]	[0.002 2.094]	[0.002 2.168]
Experiment 2, RA (RS first)	[-9.018 -3.256]	[-9.068 -2.765]	[-5.271 3.668]	[-5.760 3.493]	[0.000 3.967]	[0.000 3.827]
Experiment 2, RS (RS first)	[-7.573 -1.969]	[-7.412 -2.002]	[-6.104 1.267]	[-6.112 0.921]	[0.002 1.980]	[0.002 1.881]

Note: the numbers in each cell correspond to the 95% HDI of the posterior distribution for a particular parameter, denoting its lower and upper limit. RA = risk-averse agent; RS = risk-seeking agent

Appendix D - Choice behaviour correlations in relatively risk-averse and risk-seeking participants

The correlation analysis in the main text suggested a positive correlation in risk tendency between self decisions and predictions for the risk-average and risk-averse agents, but not the risk-seeking agent. One potential explanation is that, for most participants, the risk-seeking agent as described in the vignette may have appeared as very extreme and unrelatable, prompting them to not rely on their own decision system when making predictions for this dissimilar agent. Thus, we median-split participants based on their p_{accept} in the adaptation stage (separately for HP and LP groups) into the relatively risk-seeking and risk-averse sub-samples, and performed the correlations with predictions separately for both halves. The logic was that, if the similarity explanation holds true, then the more risk-averse subsample's behaviour should show a more positive correlation with the risk-averse agent predictions (compared to the relatively risk-seeking subsample), whereas the more risk-seeking subsample's own choices should show a more positive correlation with the risk-seeking agent predictions (compared to the relatively risk-averse subsample). The correlation results were as follows:

adaptation choices and risk-averse agent predictions:

- risk-averse subsample: rho = 0.290, p = .037) (HP); rho = 0.456, p = .001 (LP)
- risk-seeking subsample: rho = 0.419, p = .002 (HP); rho = 0.056, p = .723 (LP)

adaptation choices and risk-seeking agent predictions:

- risk-averse subsample: rho = 0.029, p = .839 (HP); rho = -0.291, p = .034 (LP)
- risk-seeking subsample: rho = -0.081, p = .567 (HP); rho = 0.025, p = .875 (LP)

In predictions for the risk-averse agent, the risk-averse HP subsample indeed showed higher correlation than the risk-seeking HP subsample. The pattern, however, was opposite in the LP group, with the risk-averse subsample showing weaker correlation than the risk-seeking sub-group. Similarly, in the predictions for the risk-seeking agent, the risk-seeking subsample showed a more positive correlation than the risk-averse subsample only in the LP group. As such, these analyses do not provide a consistently strong support for the similarity hypothesis.

Appendix E - Effects of subjective value difference/overall value

We also estimated each participant's Prospect Theory parameter values (equations 2 and 3) during each stage via maximum likelihood estimation and then used these to compute the subjective value difference (sVD), whose effect on RT we examined. Specifically, the effects of absolute sVD (the absolute difference in subjective value between the safe and risky option) and OV (a factor indicating whether the particular trial's safe option magnitude was 15 points - low - or 25 points - high) on RTs in the post-adaptation stages were assessed with the following generalised linear mixed model:

$$RT \sim\ 1 + abs(sVD) + OV + agent + risk\_group$$

The model was applied to winsorized RTs (0.05 and 0.95 percentiles), with a Gamma distribution and identity link function, to account for the non-normal distribution[1]. We note that the more complex regression models specified in the pre-registration protocol, which included random effects of intercept varying across participants, resulted in performance issues such as lack of convergence and/or singular fits in at least some conditions, leading to the use of models without interactions or random effects. To account for the interdependent nature of data in a different way, we clustered the standard errors on the level of participant for all regression tests instead[5].

For clearer interpretation of the effects of OV and sVD on RT from each condition, we also applied a similar model to each combination of agent and probability group separately. Specifically, each group was analysed with the following generalised linear model:

$$RT \sim\ 1 + abs(sVD) + OV$$

The model was applied to winsorized RTs (0.05 and 0.95 percentiles), with a Gamma distribution and identity link function, to account for the non-normal distribution.

Next, we examined whether the effect of subjective value difference (sVD) and overall value (OV) of the options was different on own decisions and predictions of others (Hypothesis 2). To this end, we fitted a linear regression model with predictors the sVD, and OV (as a factor with two levels) to response time (RT) data from each experiment's post-adaptation/prediction stage. Table S2 shows that, as is commonly observed in

Table S2

Output from the generalized linear model fitted to response time data from post-adaptation stages across experiments, sub-divided by probability group. Response times were encoded in milliseconds, and standard errors were clustered on the level of participant.

	High Probability				Low Probability			
**Predictor**	**Estimate**	**Std. error**	z	p	**Estimate**	**Std. error**	z	p
	**Control experiment, self (post-adaptation)**							
Intercept	1552.5	32.8	47.31	$< .001$	1510.8	36.4	41.55	$< .001$
$\lvert sVD \rvert$	-3.93×10^{-7}	2.39×10^{-7}	-1.65	.100	-6.03×10^{-7}	1.19×10^{-7}	-5.07	$< .001$
OV	17.84	9.53	1.87	.061	23.07	10.63	2.17	.030
	**Experiment 1, Risk-average other**							
Intercept	1475.3	35.8	41.24	$< .001$	1572.2	35.5	44.31	$< .001$
$\lvert sVD \rvert$	-1.43×10^{-7}	2.13×10^{-7}	-0.67	.500	-3.39×10^{-7}	1.41×10^{-7}	-2.41	.016
OV	7.44	10.23	0.73	.467	2.92	11.65	0.25	.802
	**Experiment 2, Risk-averse other**							
Intercept	1366.0	43.0	31.76	$< .001$	1295.7	48.6	26.68	$< .001$
$\lvert sVD \rvert$	-3.03	0.30	-10.15	$< .001$	3.00×10^{-7}	1.13×10^{-7}	2.65	.008
OV	36.72	15.26	2.41	.016	16.64	12.56	1.32	.185
	**Experiment 2, Risk-seeking other**							
Intercept	1494.1	45.4	32.92	$< .001$	1491.0	47.5	31.36	$< .001$
$\lvert sVD \rvert$	-2.97×10^{-7}	9.17×10^{-8}	-3.24	.001	-2.62×10^{-7}	9.61×10^{-8}	-2.73	.006
OV	-3.44	17.34	-0.20	.843	-18.46	14.82	-1.25	.213

decision-making tasks[6,7], higher absolute VD significantly predicted lower RT in most experiments (with the relationship not reaching significance in HP groups of the Control and Experiment 1, and having significantly positive relationship in LP of Experiment 2). Surprisingly, contrary to the established literature which finds that higher OV leads to lower RTs[8,9], in the current task it predicted higher RTs in the Control experiment; its effect was non-significant in most results of the two other experiments, with a significant positive relationship only in Risk-averse other predictions of the HP group. Overall, the effects of VD and OV are inconsistent across groups, and the individual effects often go against expectations based on previous literature. Despite this, the significant effects in the Risk-average other predictions are qualitatively the most related to the findings from the Control experiment.

In addition, we also fitted the pre-registered linear regression model with predictors of subjective value difference (sVD), factor overall value (OV), factor probability group (ProbGroupHP) and factor stage (Stage) to the response time (RT) data from each experiment separately. Table S3 shows that, as is commonly observed in decision-making tasks[6,7], higher

absolute VD significantly predicted lower RT in all three experiments. Surprisingly, higher OV either had no bearing on RT (Experiment 2) or, contrary to the established literature[8,9], predicted significantly higher RT (Control experiment, Experiment 1).

Table S3

The effects of VD and OV on RT (in milliseconds), on the full data from all three experiments.

	Control Experiment				Experiment 1				Experiment 2			
**Predictor**	**Estimate**	**Std. error**	***t***	***p***	**Estimate**	**Std. error**	***t***	***p***	**Estimate**	**Std. error**	***t***	***p***
Intercept	1564.468	35.674	43.854	$< .001$	1561.680	35.016	44.598	$< .001$	1577.758	42.147	37.435	$< .001$
$\lvert sVD \rvert$	-0.105	0.060	-1.730	.084	-0.164	0.034	-4.873	$< .001$	-0.143	0.025	-5.624	$< .001$
OV	21.173	5.611	3.774	$< .001$	11.215	5.044	2.223	.026	7.307	5.891	1.241	.215
$ProbGroup_{HP}$	105.818	48.981	2.160	.031	11.493	49.244	0.233	.815	110.737	55.584	1.992	.046
$Stage_{post-adapt}$	-81.063	15.138	-5.355	$< .001$								
$Stage_{average}$					-37.361	17.938	-2.083	.037				
$Stage_{risk-averse}$									-287.538	30.136	-9.541	$< .001$
$Stage_{risk-seeking}$									-116.172	32.971	-3.524	$< .001$

Table S4

The effects of stage, group, VD and OV on choice, fitted to the full data from all three experiments.

	Control Experiment				Experiment 1				Experiment 2			
**Predictor**	**Estimate**	**Std. error**	***t***	***p***	**Estimate**	**Std. error**	***t***	***p***	**Estimate**	**Std. error**	***t***	***p***
Intercept	-0.979	0.067	-14.707	$< .001$	-0.846	0.066	-12.886	$< .001$	-0.951	0.075	-12.628	$< .001$
$\lvert sVD \rvert$	$< .001$	$< .001$	4.705	$< .001$	$< .001$	$< .001$	6.265	$< .001$	$< .001$	$< .001$	6.837	$< .001$
OV	0.516	0.026	19.764	$< .001$	0.433	0.023	19.157	$< .001$	0.406	0.023	17.782	$< .001$
$ProbGroup_{HP}$	0.898	0.087	10.283	$< .001$	0.785	0.080	9.875	$< .001$	0.760	0.092	8.273	$< .001$
$Stage_{post-adapt}$	-0.166	0.078	-2.115	.034								
$Stage_{average}$					-0.037	0.094	-0.391	.696				
$Stage_{risk-averse}$									-1.632	0.136	-11.981	$< .001$
$Stage_{risk-seeking}$									1.277	0.124	10.295	$< .001$

Appendix F - Computational modelling

The PT-DDM consisted of six parameters - three drift rate parameters α, γ and drift scaling ds; threshold τ; non-decision time ndt; and starting point bias sp. Because of abundant individual-level data, we used a non-centered parametrisation to make the stan MCMC sampler more efficient (Papaspiliopoulos et al., 2007), whereby each of the core parameters consisted of a group-level mean μ, with an added participant-level offset z multiplied by a group-level standard deviation sd. Parameters were transformed through exponential function such that the sampling could occur on the entire real line, but the transformed parameter values would always be positive:

$par_{transformed} = log(1 + exp(mu_{par} + z_{par_{subj}} * sd_{par}))$

One notable exception was the sp parameter, whose value is strictly constrained between 0 and 1, which was achieved with the Phi transformation rather than exponentiating the combined value

The following weakly informative priors were chosen for each parameter (with parameter for each stage/group being drawn from these distributions):

$$\mu_\alpha \sim \mathcal{N}(0,1); sd_\alpha \sim h\mathcal{C}(0,0.25); z_\alpha \sim \mathcal{N}(0,1)$$

$$\mu_\gamma \sim \mathcal{N}(0,1); sd_\gamma \sim h\mathcal{C}(0,0.25); z_\gamma \sim \mathcal{N}(0,1)$$

$$\mu_{ds} \sim \mathcal{N}(0,1); sd_{ds} \sim h\mathcal{C}(0,0.5); z_{ds} \sim \mathcal{N}(0,1)$$

$$\mu_\tau \sim \mathcal{N}(0,1); sd_\tau \sim h\mathcal{N}(0,0.5); z_\tau \sim \mathcal{N}(0,1)$$

$$\mu_{ndt} \sim \mathcal{N}(0,1); sd_{ndt} \sim h\mathcal{N}(0,0.5); z_{ndt} \sim \mathcal{N}(0,1)$$

$$\mu_{sp} \sim \mathcal{N}(0,1); sd_{sp} \sim h\mathcal{C}(0,0.5); z_{sp} \sim \mathcal{N}(0,1)$$

, where $\mathcal{N}$ refers to a normal distribution and $h\mathcal{N}/h\mathcal{C}$ refers to a half-normal/half-Cauchy distribution with a lower bound on 0. The same priors and parameter specification were used for the EU-DDM/EV-DDM and heur-DDM (for the existing parameters).

To check that the PT-DDM would not provide a good account of the data if participants responded in line with a heuristic, probability-matching-like strategy, we simulated four datasets with different levels of probability matching using the heur-DDM. Each dataset

Table S5

The expected log-predictive density (ELPD) differences for the models fitted to real data.

	Control experiment				Experiment 1				Experiment 2					
	Adaptation		Post-adaptation		Adaptation		Average		Adaptation		Risk-averse		Risk-seeking	
	HP	LP	HP	LP	HP	LP	HP	LP	HP	LP	HP	LP	HP	LP
PT-DDM	0.0 (0.0)	0.0 (0.0)	0.0 (0.0)	0.0 (0.0)	0.0 (0.0)	0.0 (0.0)	0.0 (0.0)	0.0 (0.0)	0.0 (0.0)	-402.9 (578.2)	-681.1 (1064.3)	-991.5 (1525.6)	-24.4 (44.6)	0.0 (0.0)
EU-DDM	-353.9 (94.6)	-1251.6 (544.9)	-216.5 (299.0)	-944.6 (542.8)	-155.0 (58.4)	-1334.1 (221.7)	-1062.9 (351.1)	-25.0 (88.4)	-270.3 (73.3)	0.0 (0.0)	0.0 (0.0)	0.0 (0.0)	0.0 (0.0)	-73.2 (41.9)
EV-DDM	-2397.8 (328.7)	-10320.6 (2243.2)	-16363.4 (3081.7)	-17380.8 (4130.3)	-3148.2 (577.0)	-12824.5 (2924.6)	-23354.2 (6568.1)	-6694.2 (1401.1)	-3682.1 (834.9)	-19277.1 (4288.3)	-72509.4 (5569.3)	-4033531.6 (1961533.1)	-1865.8 (654.8)	-932.8 (187.3)
heur-DDM	-3012.8 (201.1)	-28955.4 (3537.0)	-12523.0 (2014.2)	-11970.0 (2584.5)	-3888.8 (453.0)	-29953.9 (3834.2)	-21805.0 (7393.8)	-6349.4 (873.7)	-4085.3 (687.0)	-33777.0 (4847.3)	-76575.9 (6181.4)	-2035891.4 (1401495.4)	-1819.2 (405.5)	-1300.4 (192.1)
**Model weights**														
PT-DDM	1.000	0.999	0.807	0.948	0.993	1.000	1.000	0.628	1.000	0.222	0.212	0.247	0.329	0.973
EU-DDM	0.000	0.001	0.193	0.052	0.007	0.000	0.000	0.372	0.000	0.778	0.788	0.753	0.671	0.027
EV-DDM	0.000	0.000	0.000	0.000	0.000	0.000	0.000	0.000	0.000	0.000	0.000	0.000	0.000	0.000
heur-DDM	0.000	0.000	0.000	0.000	0.000	0.000	0.000	0.000	0.000	0.000	0.000	0.000	0.000	0.000
**Participant model win counts**														
PT-DDM	0.516	0.350	0.500	0.436	0.496	0.511	0.574	0.394	0.587	0.389	0.510	0.411	0.365	0.305
EU-DDM	0.341	0.214	0.373	0.286	0.403	0.182	0.264	0.328	0.346	0.326	0.471	0.526	0.163	0.179
EV-DDM	0.111	0.436	0.119	0.229	0.078	0.307	0.163	0.263	0.067	0.284	0.019	0.011	0.250	0.305
heur-DDM	0.008	0.000	0.024	0.050	0.023	0.000	0.000	0.015	0.000	0.000	0.000	0.053	0.221	0.211

Note: Each cell shows the ELPD difference relative to the best-performing model, with the standard error in the parentheses. Thus, the best-performing model for a particular experimental group has an ELPD difference of 0. The next-best performing model is considered substantially worse if the magnitude of the ELPD difference is at least two times higher than the standard error of the ELPD[10]. As such, multiple models can provide a comparably good fit to the data; the cyan text indicates all such models for a particular condition.

consisted of 200 simulated participants's behaviour in the "medium probability" option set from post-adaptation stages, with each participant's parameters corresponding to the group-level parameters' mean of the posterior distribution obtained from fitting to the real data (with the drift rate being manipulated across the four datasets, to obtain the four levels of p_{accept} behaviour stated in the column headers of Table S6). We then fitted both the heur-DDM and the PT-DDM to these four datasets. As Table S6 shows, the heur-DDM provided a significantly better fit to all the simulated datasets, suggesting that participants did not, on average, employ such strategy in any of the experiments and that they did accumulate the evidence presented within trials.

Table S6

ELPD differences of the PT-DDM and heur-DDM to data generated with the heur-DDM

	Probability matching level			
	0.2	0.4	0.6	0.8
heur-DDM	0.0<br>(0.0)	0.0<br>(0.0)	0.0<br>(0.0)	0.0<br>(0.0)
PT-DDM	-10108.6<br>(78.3)	-13342.9<br>(33.6)	-12623.3<br>(36.8)	-9696.2<br>(59.6)

Figure S3

Parameter differences between the adaptation and post-adaptation/prediction stage in each experiment

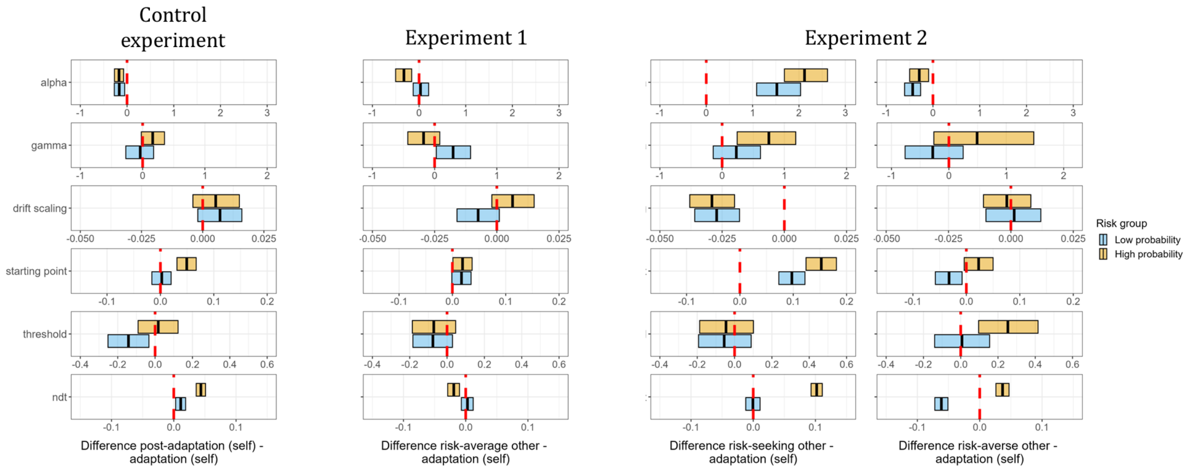

Note: Note: The thick line represents the mean of the difference, with the edges of the bars representing the 95% HDI of this distribution of difference. For practical purposes, we consider the difference to be meaningfully large if the 95% HDI does not include 0 (the dashed red line). Distributions located to the left of zero (negative values) indicate that the particular parameter had higher value in the adaptation stage, whereas distributions to the right (positive side) indicate a higher parameter value in the post-adaptation/prediction stage. As can be seen, the greatest differences (both in magnitude and in the number of affected parameters) occur in the Risk-seeking prediction stage of Experiment 2, suggesting substantially lower overlap with one's default parameters than in other experimental stages.

Table S7

Posterior group-level parameter values from the PT-DDM grouped by experiment, stage and group, for each model parameter.

	Group	μ_α	μ_γ	μ_{ds}	μ_τ	μ_{ndt}	μ_{sp}
		**Control experiment**					
Adaptation	HP	1.17<br>[1.08, 1.26]	1.11<br>[1.00, 1.22]	0.65<br>[0.64, 0.66]	2.37<br>[2.28, 2.44]	0.65<br>[0.64, 0.66]	0.43<br>[0.42, 0.44]
	LP	0.43<br>[0.42, 0.44]	1.11<br>[0.93, 1.28]	0.65<br>[0.64, 0.66]	2.36<br>[2.28, 2.44]	0.65<br>[0.64, 0.66]	0.45<br>[0.44, 0.46]
Post-adaptation	HP	0.53<br>[0.53, 0.54]	1.24<br>[1.11, 1.37]	0.88<br>[0.87, 0.88]	2.20<br>[2.12, 2.27]	0.88<br>[0.87, 0.88]	0.43<br>[0.41, 0.44]
	LP	0.42<br>[0.41, 0.44]	1.07<br>[0.95, 1.20]	0.53<br>[0.53, 0.54]	2.21<br>[2.14, 2.29]	0.53<br>[0.53, 0.54]	0.48<br>[0.47, 0.49]
		**Experiment 1**					
Adaptation	HP	1.20<br>[1.07, 1.33]	1.10<br>[0.99, 1.21]	0.62<br>[0.61, 0.63]	2.36<br>[2.28, 2.46]	0.43<br>[0.42, 0.44]	0.46<br>[0.45, 0.47]
	LP	1.25<br>[1.15, 1.35]	1.16<br>[0.98, 1.35]	0.62<br>[0.61, 0.63]	2.36<br>[2.28, 2.46]	0.42<br>[0.42, 0.43]	0.47<br>[0.46, 0.48]
Risk-average	HP	0.66<br>[0.65, 0.67]	1.28<br>[1.11, 1.45]	0.66<br>[0.65, 0.67]	2.17<br>[2.10, 2.26]	0.66<br>[0.65, 0.67]	0.47<br>[0.46, 0.48]
	LP	0.42<br>[0.42, 0.43]	1.46<br>[1.27, 1.64]	0.66<br>[0.65, 0.67]	2.25<br>[2.17, 2.33]	0.25<br>[0.25, 0.26]	0.46<br>[0.45, 0.48]
		**Experiment 2**					
Adaptation	HP	1.62<br>[1.56, 1.70]	1.09<br>[0.98, 1.18]	0.59<br>[0.58, 0.60]	2.62<br>[2.51, 2.74]	0.59<br>[0.58, 0.60]	0.45<br>[0.44, 0.47]
	LP	0.56<br>[0.55, 0.56]	1.40<br>[1.13, 1.67]	0.55<br>[0.55, 0.56]	2.59<br>[2.50, 2.73]	0.55<br>[0.55, 0.56]	0.42<br>[0.41, 0.44]
Risk-averse	HP	0.33<br>[0.31, 0.36]	1.61<br>[0.95, 2.38]	0.09<br>[0.08, 0.09]	2.59<br>[2.50, 2.73]	0.43<br>[0.42, 0.44]	0.42<br>[0.40, 0.43]
	LP	0.36<br>[0.30, 0.42]	1.12<br>[0.74, 1.54]	0.09<br>[0.08, 0.10]	2.34<br>[2.24, 2.46]	0.47<br>[0.46, 0.47]	0.40<br>[0.37, 0.42]
Risk-seeking	HP	1.83<br>[1.66, 2.10]	1.87<br>[1.67, 2.09]	0.06<br>[0.05, 0.07]	2.33<br>[2.23, 2.39]	1.24<br>[1.23, 1.25]	0.56<br>[0.54, 0.58]
	LP	1.90<br>[1.66, 2.13]	1.63<br>[1.39, 1.88]	0.06<br>[0.05, 0.07]	2.29<br>[2.20, 2.39]	0.51<br>[0.50, 0.51]	0.53<br>[0.51, 0.55]

Note: the number in each cell corresponds to the mean of the posterior distribution for a particular group-level parameter, with the brackets showing the upper and lower value of the distribution's 95% HDI. Note that the parameters were originally sampled on a different scale, but the table shows the re-transformed values for easier interpretation.

Figure S4

Posterior predictive check of the PT-DDM on response times

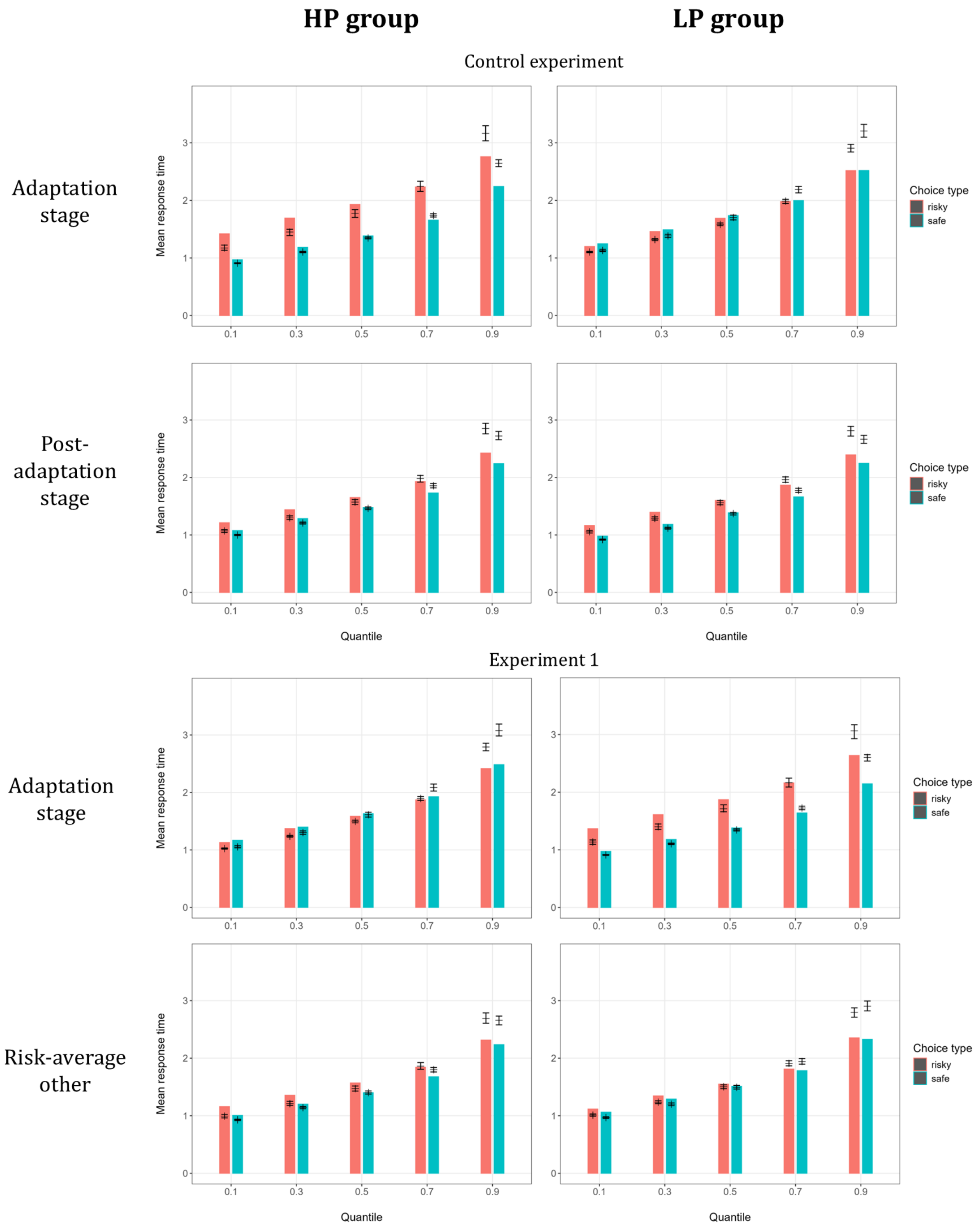

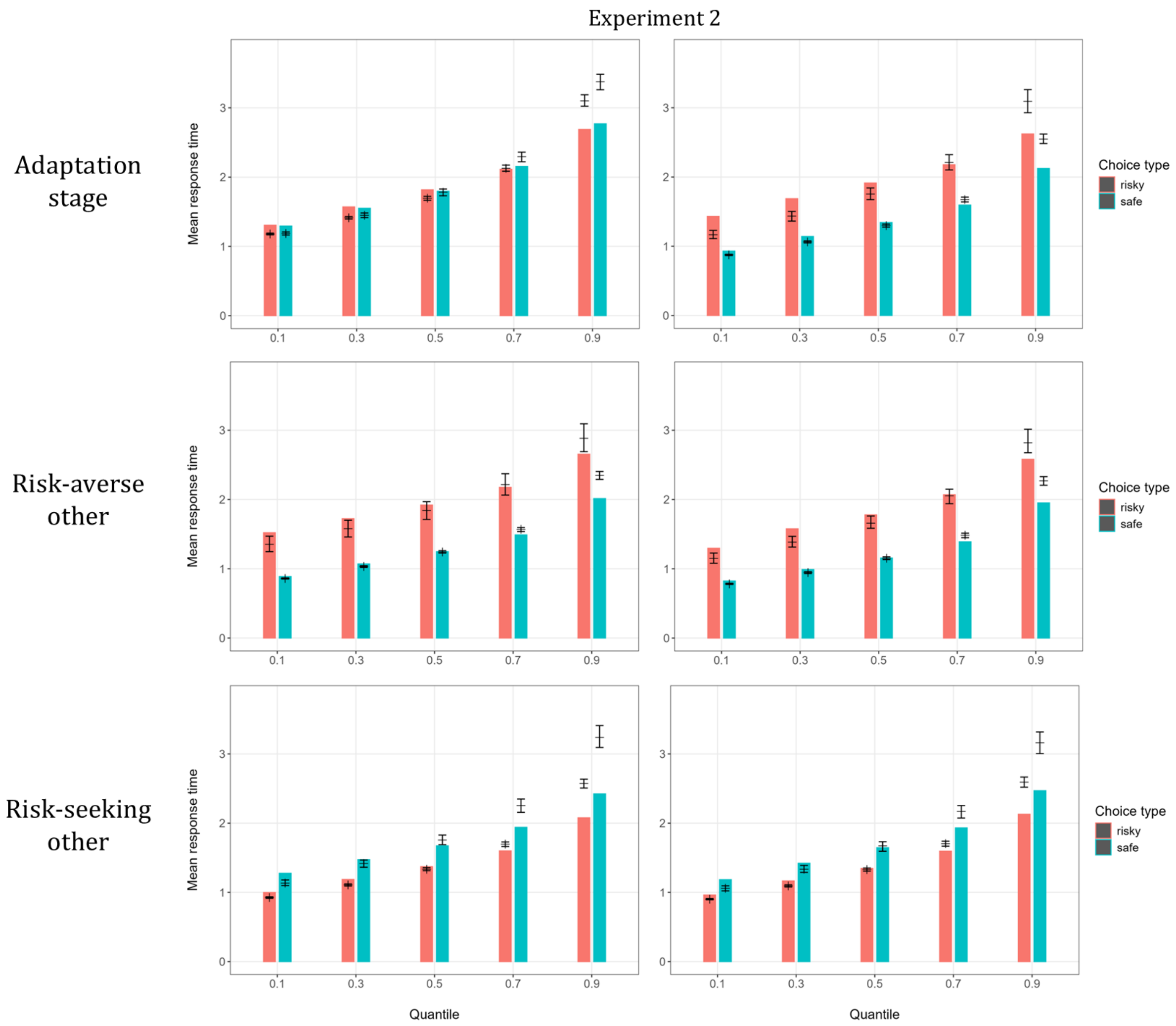

Note: Each panel displays the 5 quantile means of the response time distribution for the particular condition as bars, split by risky and safe choices. The super-imposed black lines with whiskers show the mean and 95% HDI of the group-level mean safe/risky RTs obtained from 500 simulated datasets, all generated with the best-fitting parameter values in that condition (i.e., the posterior draw with the highest negative log likelihood value was used to determine the participant-level parameters used for simulations).

Figure S5

Participant-level posterior predictive check of the PT-DDM on choice and response times.

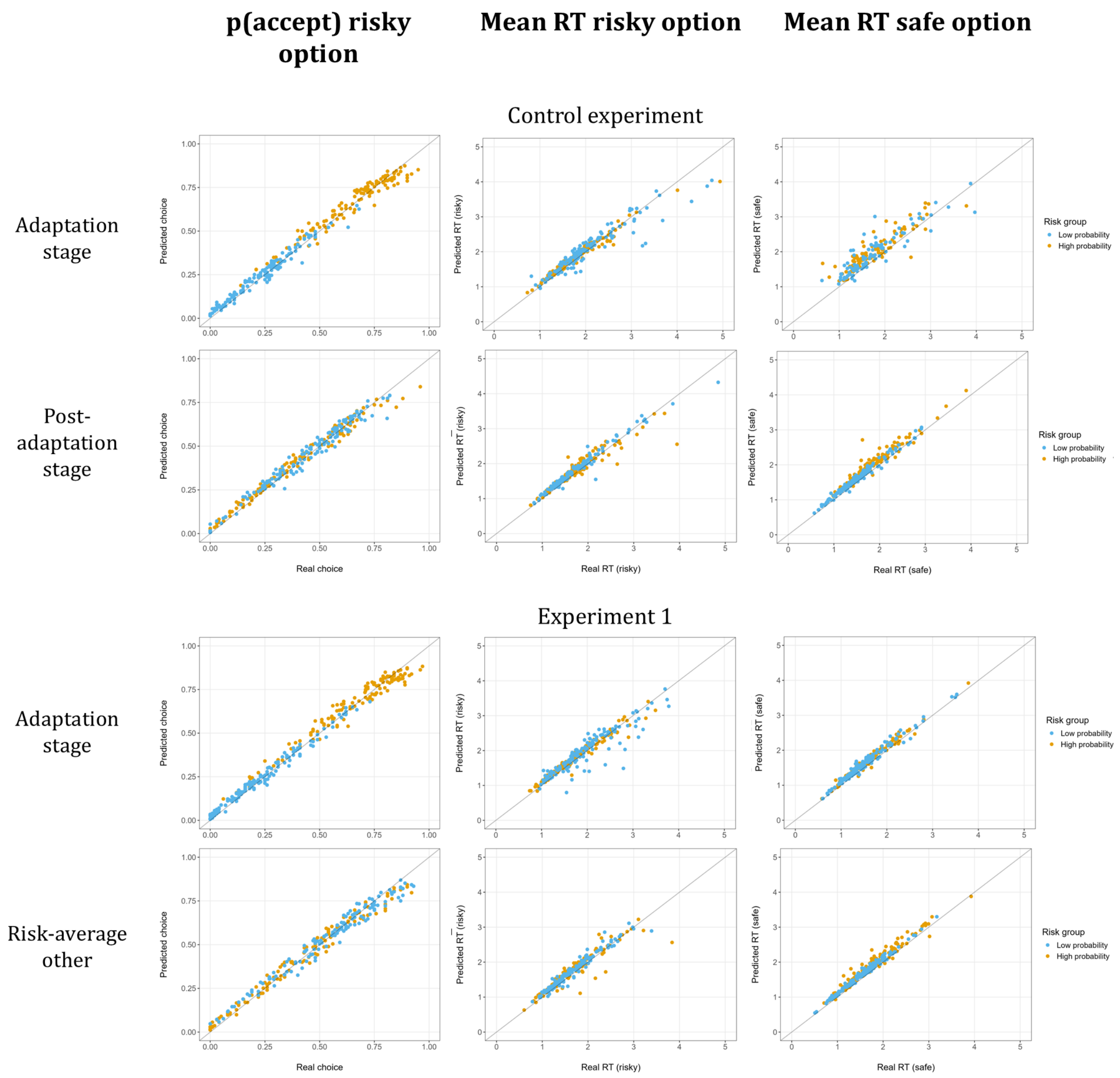

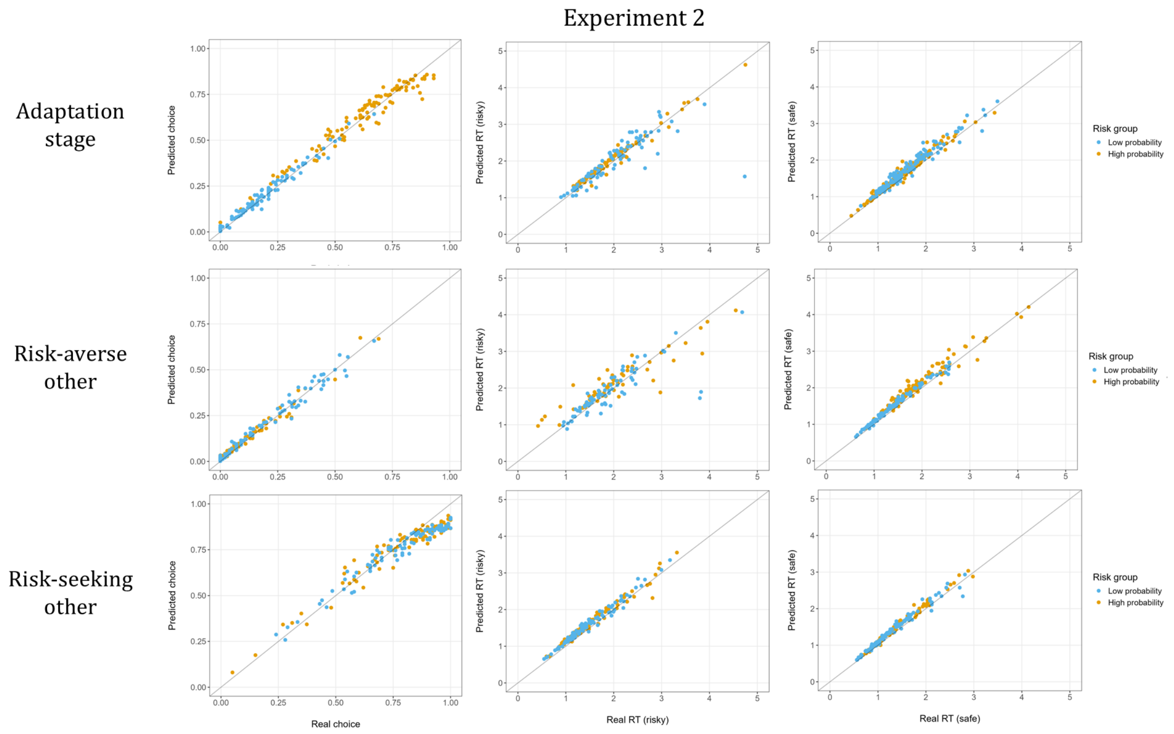

Note: The x-axis represents the participant's real mean choice/RT in the given condition, with the y-axis displaying the participant-level mean from across the 500 simulated datasest. Each point represents a single participant.

Table S8

The expected log-predictive density (ELPD) differences for three versions of the PT-DDM

	Control experiment				Experiment 1				Experiment 2					
	Adaptation		Post-adaptation		Adaptation		Average		Adaptation		Risk-averse		Risk-seeking	
	HP	LP	HP	LP	HP	LP	HP	LP	HP	LP	HP	LP	HP	LP
PT-flexible	-461.2 (150.6)	-6730.2 (802.8)	-3724.6 (1513.2)	-1290.3 (860.4)	-219.2 (157.9)	-7312.6 (1025.8)	-1488.6 (560.1)	-1425.5 (459.1)	-1102.2 (369.3)	-8479.4 (1773.1)	-30564.2 (4493.8)	-33153.3 (6462.8)	0 (0.0)	0.0 (0.0)
PT-semi-static	-401.7 (131.2)	-696.7 (999.4)	-2155.5 (393.9)	0.0 (0.0)	0.0 (0.0)	-3504.5 (467.2)	-3866.4 (1488.7)	-788.7 (396.5)	-1218.5 (336.2)	-6022.8 (992.0)	-5168.1 (1553.4)	-12773.9 (2511.0)	-121.4 (191.4)	-253.2 (80.3)
PT-static	0.0 (0.0)	0.0 (0.0)	0.0 (0.0)	-52.8 (1092.4)	-50.2 (218.7)	0.0 (0.0)	0.0 (0.0)	0.0 (0.0)	0.0 (0.0)	0.0 (0.0)	0.0 (0.0)	0.0 (0.0)	-5527.3 (359.4)	-3928.1 (340.2)
**Model weights**														
PT-flexible	0.000	0.000	0.000	0.000	0.026	0.000	0.002	0.000	0.000	0.000	0.000	0.000	0.277	0.999
PT-semi-static	0.000	0.202	0.000	0.442	0.609	0.000	0.000	0.002	0.000	0.000	0.000	0.000	0.723	0.001
PT-static	1.000	0.798	1.000	0.558	0.364	1.000	0.998	0.998	1.000	1.000	1.000	1.000	0.000	0.000
**Participant model win counts**														
PT-flexible	0.310	0.086	0.325	0.314	0.302	0.146	0.380	0.307	0.346	0.137	0.087	0.158	0.596	0.663
PT-semi-static	0.198	0.250	0.119	0.207	0.279	0.095	0.140	0.255	0.154	0.074	0.375	0.221	0.317	0.232
PT-static	0.492	0.664	0.556	0.479	0.419	0.759	0.481	0.438	0.500	0.789	0.538	0.621	0.087	0.105

Note: we adapted the fully flexible version of the model (the hierarchical PT-DDM) and compared its fit against two, more restricted variants: the "static" PT-DDM whereby all parameters are fixed across the adaptation and post-adaptation stage (except for the non-decision time, which has shown large between-stage variance in original model fits and as such, any model which fixes the value of this parameter is unlikely to perform well); and the "semi-static" PT-DDM whereby alpha and starting point bias are also free to vary across the stages - the logic being that any form of simulation hypothesis would likely include one/both of these parameters.

Figure S6

Group-level parameter recovery for the PT-DDM

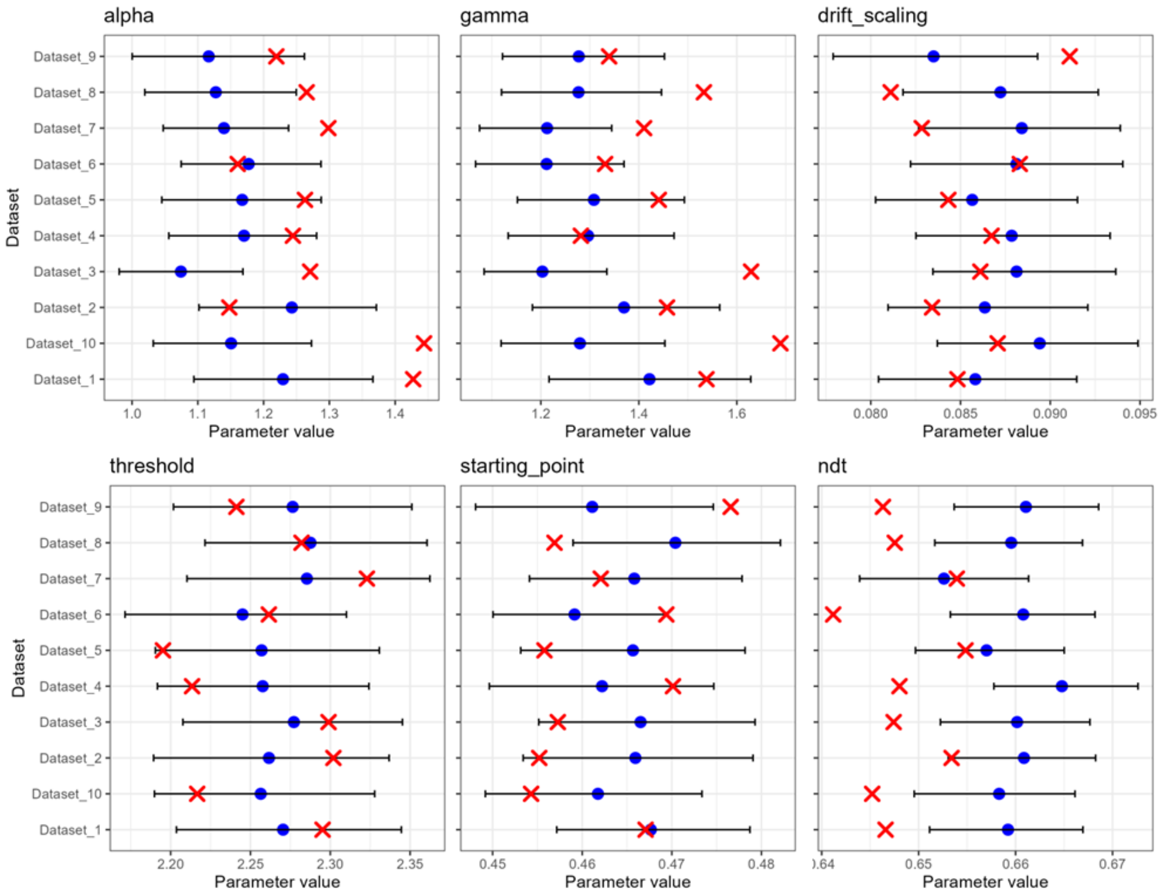

Note: Each row represents a single simulated dataset, which was based on options and parameters obtained Experiment 1 (from all stages and groups). Parameters for each dataset were selected by rank-ordering the posterior distrubiton draws from the most to the least likely (in terms of the highest log likelihood), and then simulating a decision set with the individual-level parameters for each of the 10 most likely posterior draws. The red cross represents the generative group-level parameter value for the particular posterior draw, and the blue circle displays the recovered mean of the parameter's posterior distribution, with the whiskers representing the 95% HDI of this distribution. The recovery can be deemed successful if the HDI encompasses the generative value signified by the red cross.

Figure S7

Posterior predictive check for the static and semi-static versions of the PT-DDM

A)

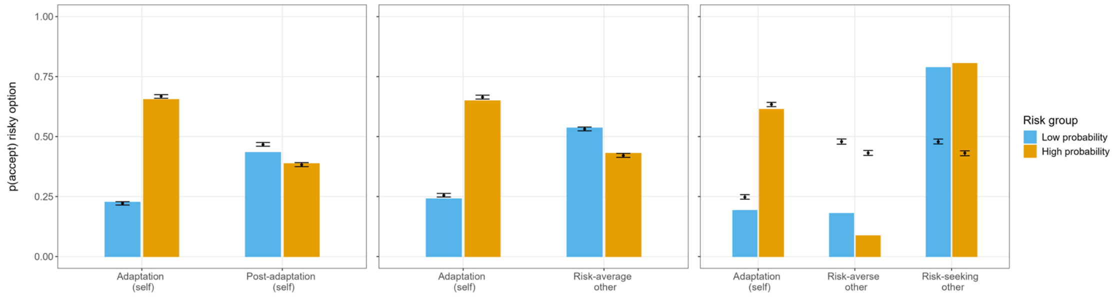

B)

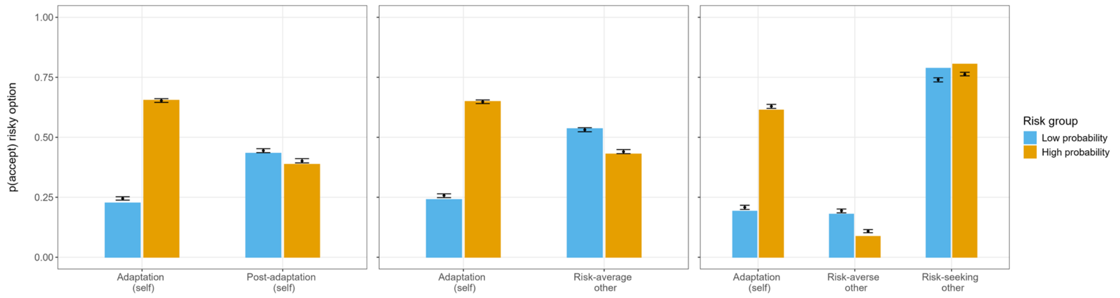

Note: The bars show the real group-level choice data in each experimental stage. The super-imposed black points with whiskers show the mean and 95% HDI of the mean choices obtained from 500 simulated datasets, all generated with the best-fitting parameter values in that condition (i.e., the posterior draw with the highest negative log likelihood value was used to determine the participant-level parameters used for simulations). A) The static PT-DDM; B) the semi-static PT-DDM

Supplementary references

[1] Lo, S., & Andrews, S. (2015). To transform or not to transform: Using generalized linear mixed models to analyse reaction time data. *Frontiers in Psychology*, *6*. https://doi.org/10.3389/fpsyg.2015.01171

[2] Lenth, R. V. (2025). *Emmeans: Estimated marginal means, aka least-squares means* [R package version 1.11.2-80003]. https://rvlenth.github.io/emmeans/

[3] Liu, Q., & Wang, L. (2021). T-test and ANOVA for data with ceiling and/or floor effects. *Behavior Research Methods*, *53*(1), 264–277. https://doi.org/10.3758/s13428-020-01407-2

[4] Bürkner, P.-C. (2017). Brms: An r package for bayesian multilevel models using stan. *Journal of Statistical Software*, *80*, 1–28. https://doi.org/10.18637/jss.v080.i01

[5] Abadie, A., Athey, S., Imbens, G. W., & Wooldridge, J. M. (2023). When should you adjust standard errors for clustering?*. *The Quarterly Journal of Economics*, *138*(1), 1–35. https://doi.org/10.1093/qje/qjac038

[6] Roe, R. M., Busemeyer, J. R., & Townsend, J. T. (2001). Multialternative decision field theory: A dynamic connectionst model of decision making [Place: US Publisher: American Psychological Association]. *Psychological Review*, *108*(2), 370–392. https://doi.org/10.1037/0033-295X.108.2.370

[7] Teodorescu, A. R., Moran, R., & Usher, M. (2016). Absolutely relative or relatively absolute: Violations of value invariance in human decision making. *Psychonomic Bulletin & Review*, *23*(1), 22–38. https://doi.org/10.3758/s13423-015-0858-8

[8] Ting, C.-C., & Gluth, S. (2023). High overall values mitigate gaze-related effects in perceptual and preferential choices [Publisher: OSF]. https://doi.org/10.31234/osf.io/dvj7z

[9] Pirrone, A., Azab, H., Hayden, B. Y., Stafford, T., & Marshall, J. A. R. (2018). Evidence for the speed-value trade-off: Human and monkey decision making is magnitude sensitive. *Decision (Washington, D.C.)*, *5*(2), 129–142. https://doi.org/10.1037/dec0000075

[10] Vehtari, A., Gelman, A., & Gabry, J. (2017). Practical bayesian model evaluation using leave-one-out cross-validation and WAIC. *Statistics and Computing*, *27*(5), 1413–1432. https://doi.org/10.1007/s11222-016-9696-4
